# Research on the construction of an indicator system for physical education teaching abilities of preschool teachers

**DOI:** 10.3389/fpsyg.2025.1674552

**Published:** 2025-12-16

**Authors:** Zuozheng Shi, Xulin Yang, Binglong Xu, Xi Long

**Affiliations:** 1 Early Childhood Sports and Health Research Centre, Chongqing Preschool Education College, Chongqing, China; 2 Mental Health Counseling Center, Chongqing Preschool Education College, Chongqing, China

**Keywords:** early childhood teachers, early childhood sports, early childhood physical education curriculum, Delphi method, structured physical education curriculum

## Abstract

**Background:**

The purpose of this study is to construct a physical education competency index system for early childhood teachers to improve their practical ability to structure early childhood physical education programs and to promote the high quality development of early childhood physical education programs.

**Methods:**

Using the Delphi method, a multidisciplinary expert group was formed to systematically evaluate the physical education competency index system for early childhood teachers. The consistency and stability of the physical education competency index system for preschool teachers were investigated in detail using a 5-point Likert scale.

**Results:**

After two rounds of Delphi surveys, the results showed that consensus was reached on all 5 first-level indicators, 17 s-level indicators, and 54 third-level indicators included in the physical education competency indicator system for preschool teachers, and that the stability of the indicators was good. The physical education competency index system we have developed for early childhood teachers is reliable and reasonable.

**Conclusion:**

The findings of this study not only effectively enhance the physical education teaching skills of preschool teachers, but also provide a basis for the development and evaluation of structured physical education curricula for preschoolers.

## 1 Introduction

In recent years, UNESCO has proposed “Quality Physical Education” as part of school sports reform. It is defined as a planned, progressive, and inclusive learning experience within preschool, primary, and secondary education curricula, serving as the foundation for lifelong participation in physical activity and sports (Bao, 2017). Regarding the development of children and adolescents’ health, China proposed a child-first development strategy in the Healthy Children Action Plan (2018–2020), with the promotion of children’s health listed as the top priority action (National Health Commission of the People’s Republic of China, 2018). Regarding the development of preschool children, China’s Outline for Building a Strong Nation in Sports proposes to improve policies and safeguard systems for early childhood physical education, vigorously advance the development of early childhood sports programs and standards for sports equipment, and establish a curriculum system and teacher training system for early childhood physical education (General Office of the State Council, 2019). The Outline of the Plan for Building China into an Education Powerhouse (2024–2035) states that efforts should be intensified to implement the project for enhancing the quality and excellence of basic education, promoting students’ healthy growth and all-round development (Ministry of Education of the People’s Republic of China, 2025a). The Law of the People’s Republic of China on Preschool Education explicitly stipulates that kindergartens shall base their programs on the daily lives of preschool children, with play as the primary activity. They shall advance quality education, maximally supporting preschoolers in exploring and learning through close interaction with nature, hands-on activities, and direct experiences. This approach fosters the development of sound moral character, behavioral habits, safety awareness, and work ethic among preschool children, cultivating well-rounded personalities and robust physical health. and achieve balanced development across health, language, social skills, science, and arts (Ministry of Education of the People’s Republic of China, 2025b). These policy guidelines aim to provide children and adolescents with age-appropriate learning experiences through physical education programs, helping them acquire essential motor skills, cognitive abilities, physical literacy, and social–emotional skills. This provides a crucial foundation for the present study.

Preschool children refer to those aged 3 to 6 years old, specifically the group that has not yet entered elementary school and is currently in the kindergarten education stage. Early childhood teachers are the primary providers of care and education for preschool children aged 3 to 6 years old. Through physical education, young children learn fair competition, overcome fears, and cultivate qualities of courage, decisiveness, and perseverance. These foundational skills play a fundamental role in enhancing their subsequent learning abilities, critical thinking, and social interaction skills (Zhang et al., 2021). The promotion of young children’s attention, attitudes, behaviors, and academic performance is directly proportional to the amount of daily physical activity they engage in (Tang and Qin, 2016; Nagel, 2016; Greenspan, 2000). It is also the most direct way to influence their basic motor skills and physical fitness. These studies indicate that physical education, as an essential component of early childhood education, plays an irreplaceable role in early education.

Children aged 3–6 years old are in a critical period for establishing and developing fundamental motor skills (Clark and Metcalfe, 2002; Lubans et al., 2010). These basic motor skills serve as the building blocks for physical literacy, which can be effectively and comprehensively cultivated through physical education. A lack of exposure to and practice of fundamental motor skills during this age range will directly impact children’s future participation levels in physical activities (Hai, 2021). Children’s fundamental motor skills do not develop naturally; they require instruction, practice, and reinforcement within appropriate movement patterns to be acquired (Robinson, 2011a; Robinson, 2011b; Logan et al., 2012; Robinson et al., 2012). Organized, structured curricula significantly enhance basic motor skill development compared to unstructured programs (Abusleme-Allimant et al., 2023; Zhou et al., 2021). Furthermore, physical activities within structured curricula foster a greater sense of educational belonging (Côté et al., 2003). These research findings reveal the necessity of developing and implementing structured physical education curricula for young children.

Current research indicates that early childhood educators lack sufficient understanding of the value and principles of physical education (Dan, 2017). Goals for physical education in early childhood settings remain unclear, and educators in this field exhibit inadequate professional competence (Zhang, 2023). Preschool teachers lack sufficient experience in organizing and teaching physical activities, with weak foundational knowledge and practical skills in physical education (Chunying, 2020). Preschool teacher training programs fail to cultivate physical education teaching competencies aligned with early childhood education, with curriculum designs lacking a holistic development perspective. Teaching methods remain outdated and monotonous, while assessment systems are inadequate (Huawei et al., 2021). The implementation of low-intensity, self-selected, and unstructured outdoor activities in kindergartens may contribute to insufficient exercise effects, potentially delaying the development of basic motor skills. There is an urgent need for moderate-to-high intensity, structured physical activity approaches (Ning et al., 2022). Therefore, addressing these issues requires systematic research on the physical education competencies of early childhood educators.

In case studies examining factors influencing physical education for preschool children, the teacher’s ability to teach motor skills is one of the most influential factors (Marinsek and Kovac, 2019). Fundamental movement skills result from the combined effects of multiple factors such as “practice, encouragement, instruction, and an appropriate learning environment,” requiring careful teaching by educators (Shape, 2014). Developing motor competence and physical confidence is a process that necessitates appropriate feedback, guidance, and organized motor skill learning (MacNamara et al., 2015). Insufficient confidence levels and weak knowledge of physical education.

content among early childhood educators are key barriers to “effective physical education instruction” (Vives-Rodriguez, 2005). Early childhood educators need to know how to design and implement developmental learning experiences in physical education (Tsangaridou, 2017). Their content knowledge and pedagogical knowledge should be analyzed within the context of structured physical activity programs (Martínez-Bello et al., 2020). Furthermore, the utilization of curriculum planning, resources, and equipment within educational institutions should be integrated as part of teacher professional development (Jones et al., 2019). These research findings provide a reference for selecting physical education competency indicators for early childhood teachers.

Research suggests that preschool physical activity interventions that target teacher-led strategies can have the highest beneficial outcomes for children’s physical activity levels (Webster et al., 2020). Early childhood educators’ physical literacy plays a crucial role in promoting children’s physical motor development, enhancing their awareness of lifelong physical activity, and fostering their socialization (Chang, 2019). Teachers’ professional development positively impacts young children’s learning and development (Jensen and Rasmussen, 2018). Early childhood educators play a supportive and guiding role in children’s physical activities, and their physical education competency directly influences the implementation and effectiveness of such activities (Wang et al., 2025). Teacher-guided and teacher-led indoor preschool physical activities can increase moderate-to-vigorous physical activity levels among preschool children (Carroll et al., 2022). It is recommended to design thematic curricula emphasizing foundational motor skills and physical activity behaviors. This approach not only fosters preschoolers’ understanding of active lifestyles but also reinforces academic knowledge (e.g., counting, body awareness) (Webster et al., 2020). These studies indicate that early childhood educators’ physical education competencies directly determine the effectiveness of children’s participation in physical activities.

Although numerous studies have been conducted on the core physical education competencies and teaching abilities of early childhood educators, such as: the Physical Education Competency Indicator System for Early Childhood Educators (Xu, 2023), the Evaluation Indicator System for Physical Education Teaching Competencies of Early Childhood Educators (Shao, 2021; Keyuan, 2022), Professional Quality Indicator System for Early Childhood Physical Education Teachers (Kaiheng, 2025), Diagnostic Evaluation System for Physical Education Competency in Early Childhood Teachers (Jin, 2022; Lu, 2023). However, few studies have systematically and practically explored early childhood teachers’ physical education competencies grounded in structured physical education curricula. Therefore, we have fully incorporated the physical education model for regional physical activities in kindergartens (Daryl, 1994) and the Physical Fitness and Motor Ability or Sport Game (PMS) model (Cao, 2023). We have attempted to develop a structured physical education curriculum assessment tool for kindergarten teachers based on structured teaching and practice. This tool aims to enhance the quality of physical education instruction and learning for young children while promoting teachers’ professional development. The development of a competency framework for physical education in early childhood education offers several advantages. First, it integrates physical education concepts into early childhood education, enabling systematic research on preschool teachers’ physical education competencies. Second, enhancing early childhood educators’ self-awareness and practical skills in physical education curricula will effectively promote the development and implementation of structured physical education programs for young children. Finally, this assessment system may enhance the development of young children’s basic motor skills and physical fitness.

## 2 Materials and methods

**Figure 1:**
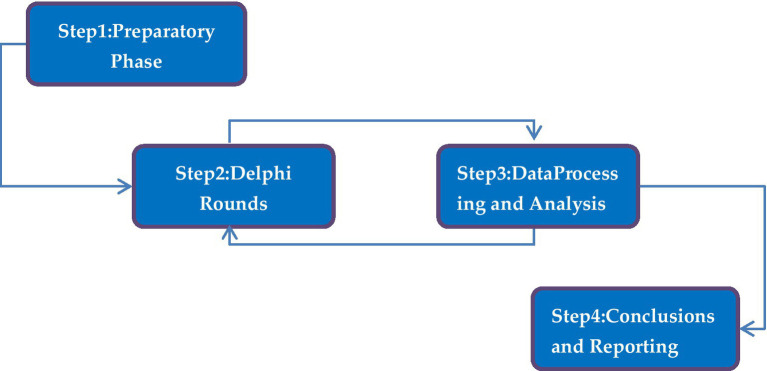
The Delphi research path.

Delphi is a method of obtaining expert opinions to support, challenge, summarize, and verify consensus (Yan et al., 2021). This study adopted the modified Delphi technique to organize, collect, and provide information related to the professional field in order to obtain consensus among experts in the field (Sharkey and Sharples, 2001; Denecke et al., 2024). We conducted keyword searches for “early childhood physical education” and “early childhood physical education curriculum” in the Web of Science, Scopus, CNKI, and Google Scholar databases. Drawing upon research findings, current status and development trends from multiple developed nations including the United States (Marika et al., 2021; Adams and Wolf, 2008; Xia and Hui, 2017), Japan (Figueroa et al., 2019), Germany (Blömeke, 2019), the United Kingdom (Liu and Zhang, 2019), Italy (D'elia et al., 2018), Canada (Lu and Lodewyk, 2012). this study has preliminarily established an indicator system for early childhood educators’ physical education competencies. This framework integrates structured physical education curriculum design with practical teaching methodologies, including 5 first-level indicators, 18 s-level indicators, and 63 third-level indicators, as shown in Appendix 1. The preliminary indicator system was implemented after conducting interviews with five early childhood educators to ensure the research content was comprehensible within current academic research and practice. The Delphi method is used to collect and apply data: The first step is to select experts, establish criteria for their inclusion, and form a panel of experts. The second step is to conduct the survey and interview experts in the first and second rounds. The third step is data processing and analysis, which involves quantitative and qualitative analysis of the survey results. The fourth step is to report the results and conclusions, discuss the findings of the investigation, and draw conclusions from the research. Figure 1 shows the research process.

### 2.1 Selection of experts

The number of experts selected for the Delphi method varies depending on the scope of the study, but it is generally recommended to select between 8 and 20 experts (Jian-Yu and Xiao-Qiu, 2023), some guidelines have suggested the involvement of 15 participants (McMillan et al., 2016). The selection of experts is an important criterion for the effectiveness of Delphi research (Aghimien et al., 2020), and the results mainly depend on the insights and opinions of the expert panel. As this is an interdisciplinary study spanning early childhood education and sports science, we recruited scholars nationwide with backgrounds in both sports science and early childhood education, who possess teaching and research experience in early childhood physical education curricula. After conducting an in-depth assessment of candidates’ academic backgrounds and practical experience, the research team established selection criteria based on research background, professional experience, and teaching and administrative expertise. The inclusion criteria are as follows: (1) Assistant professors who have been engaged in early childhood education and physical education teaching for more than 10 years and have at least a bachelor’s degree; (2) teachers with at least a master’s or doctoral degree who have been engaged in early childhood education and physical education for more than 5 years; (3) at least 5 years of experience as a manager in kindergarten teaching and research. Table 1 for expert profiles.

**Table 1:** Summary of experts characteristics.

Characteristics	Round 1 (*n* = 18), *n* (83.33%)	Round 2 (*n* = 15), *n* (100%)
Gender		
Male	9 (60.00%)	9 (60.00%)
Female	6 (40.00%)	6 (40.00%)
Total	15	15
Academic qualifications		
Bachelor’s degree	9 (60.00%)	9 (60.00%)
Master’s degree	4 (26.67%)	4 (26.67%)
Ph.D. degree	2 (13.33%)	2 (13.33%)
Total	15	15
Field of research		
Kinesiology	7 (46.66%)	7 (46.66%)
Pre-primary education	8 (15.34%)	8 (15.34%)
Total	15	15
Years of experience		
5 years	1 (6.66%)	1 (6.66%)
6–10 years	3 (20.00%)	3 (20.00%)
11–15 years	5 (33.33%)	5 (33.33%)
Over 15 years	6 (40.00%)	6 (40.00%)
Total	15	15
Working organization		
Research organization (College)	12 (80.00%)	12 (80.00%)
Early education organization	3 (20.00%)	3 (20.00%)
Total	15	15

### 2.2 Delphi rounds

The Delphi method was used to explore the indicator system for physical education capabilities of preschool teachers. The preliminary indicator system was tested by a group of experts. Based on the test results, discussions and revisions were made, and a questionnaire survey was conducted. The survey was concluded after all experts reached a consensus. In each round of surveys, we carefully analyze the opinions of the expert panel, listen to their suggestions and feedback, and optimize the survey tools by adding, deleting, merging, or modifying them. We carry over all questions from each round of questionnaires to the next round of surveys, including the consensus reached in the previous round of surveys. We define “Consensus” as more than 75% of researchers giving a score of 4 or 5, indicating agreement with the view (Denecke et al., 2024). In each round of surveys, the expert panel has the opportunity to change their views, which are used as a basis for calculating the stability of the survey results until consensus is reached on the survey content. By compiling and providing feedback on the first round of recommendations, and identifying the consensus and points of disagreement among experts; the second round highlights these points of disagreement, prompting experts with differing viewpoints to re-evaluate or supplement their arguments, thereby narrowing the gaps. Since the development of an indicator system for early childhood teachers’ physical education capabilities is an interdisciplinary research project, we primarily used closed-ended questions and supplemented them with open-ended questions for our survey. Closed-ended questions provide a basis for quantitative analysis, while open-ended questions enable expert panels to express forward-looking and innovative views.

### 2.3 Data procession and analysis

The data were analyzed using Excel 2024 and SPSS 29.0 software for descriptive statistics. A 5-point Likert scale was employed to evaluate the data by assigning scores from 1 to 5, ranging from “unimportant” to “very important.” The interquartile range (IQR) of each 5-point Likert question response were calculated. We followed the recommendations of Vonder Gracht (2012) for finding consensus. In this regard, we considered that agreement with an item was reached when the IQR of the participants’ responses to this item in the round was ≤1. The IQR is usually found to be a suitable consensus criterion for 4- or 5-unit scales. Following this criterion, we defined “agreement” with an item in a given round as the IQR of the participants’ responses being ≤1 and defined “disagreement” otherwise. As it is recommended by Vonder Gracht (2012) we also defined the stability between rounds as follows. Participants’ responses to an item in 2 consecutive rounds were considered stable when the median of these responses failed to show a statistically significant difference between the rounds. We used the Wilcoxon matched-pairs signed rank test to assess the stability in these responses. This test is commonly used to assess the stability of responses in 2 consecutive rounds in Delphi studies. Following these criteria, we considered that participants’ responses to an item in 2 consecutive rounds were stable when the results of the Wilcoxon matched-pairs signed rank test did not show a statistically significant difference and considered them unstable otherwise. The authority of experts is calculated using the formula Cr = (Cs + Ca)/2. The higher the score, the more authoritative the expert opinion (Chen et al., 2022). In addition, the 1–9 scale method proposed was used, with experts conducting pairwise comparisons and scoring to determine the relative importance of evaluation indicators at the same level (Saaty, 1987). The consistency of the indicators was tested using CR < 0.01 as the test standard.

### 2.4 Conclusion and reporting

Report our research results in the discussion and conclusion sections, including the assessment, consensus, stability, and weight values of the indicator system.

## 3 Results

### 3.1 Round one

In the first round of the survey, we sent out 18 invitations, and 16 experts participated in the survey and responded. There was one invalid questionnaire, and the effective response rate was 83.33%. The rate of expert modification suggestions was 70%, the expert panel’s judgment basis was Ca = 0.91, familiarity was Cs = 0.87, and authority was Cr = 0.89. The validity and reliability of the research tool were determined through Cronbach’s alpha testing (*α* = 0.982), indicating that the research tool has high validity and reliability. The survey content is shown in Appendix 1. The first round of Delphi surveys collected the results of the expert panel on the indicator system for physical education capabilities of early childhood teachers, as shown in Table 2. In the first round of Delphi survey results, the expert panel reached consensus on five first-level indicators, 17 s-level indicators, and 51 third-level indicators, and proposed modifications to the survey indicators. We summarized common issues raised by the expert panel and clarified and revised the survey indicators.

**Table 2:** Results of round one of the Delphi study.

Indexes	Agreement or disagreement	Scores of 4 or 5	Scores of 5	Results
A. Basic literacy	Agreement	14 (93.3)	11 (73.3)	Reservation
A1. Physical fitness	Agreement	13 (86.7)	13 (86.7)	Reservation
A1-1: Healthy fitness	Agreement	15 (100)	12 (80)	Reservation
A1-2: Athletic fitness	Agreement	15 (100)	11 (73.3)	Reservation
A2. Motor skills	Agreement	15 (100)	12 (80)	Reservation
A2-1: Mobility skills	Agreement	15 (100)	11 (73.3)	Reservation
A2-2: Manipulative skills	Agreement	15 (100)	10 (66.7)	Reservation
A2-3: Stability skills	Agreement	15 (100)	12 (80)	Reservation
A3. Health behaviors	Agreement	13 (86.6)	11 (73.3)	Reservation
A3-1: Health perceptions	Agreement	14 (93.3)	11 (73.3)	Reservation
A3-2: Healthy habits	Agreement	13 (86.7)	9 (60)	Reservation
A3-3: Emotional control	Agreement	15 (100)	9 (60)	Reservation
A4. Sportsmanship	Agreement	14 (93.3)	12 (80)	Reservation
A4-1: Movement confidence	Agreement	14 (93.3)	11 (73.3)	Reservation
A4-2: Follow the rules	Agreement	14 (93.3)	10 (66.7)	Reservation
A4-3: Fair play	Agreement	15 (100)	11 (73.3)	Reservation
A4-4: Solidarity	Agreement	15 (100)	11 (73.3)	Reservation
A5. Sports science knowledge	Agreement	14 (93.3)	12 (80)	Reservation
A5-1: Basic terminology in physical education	Disagreement	14 (93.3)	10 (66.7)	Remove
A5-2: Characteristics of early childhood kinesiology	Agreement	14 (93.3)	10 (66.7)	Revise
A5-3: Patterns of early childhood physical and Mental development	Disagreement	14 (93.3)	11 (73.3)	Remove
A5-4: Early childhood physical education values	Disagreement	12 (80)	10 (66.7)	Remove
A5-5: Early childhood physical education goals	Disagreement	14 (93.3)	9 (60)	Remove
A5-6: Early childhood physical education methods	Disagreement	15 (100)	11 (73.3)	Remove
A5-7: Early childhood physical education content	Disagreement	14 (93.3)	10 (66.7)	Remove
A5-8: Evaluation of early childhood physical education	Disagreement	15 (100)	10 (66.7)	Remove
A5-9: Structured physical activity design	Agreement	15 (100)	11 (73.3)	Reservation
A5-10: Interdisciplinary integration curriculum design	Agreement	14 (93.3)	10 (66.7)	Reservation
A5-11: Making your own playthings and creating games	Disagreement	14 (93.3)	11 (73.3)	Remove
A5-12: Early childhood sports protection and handling	Agreement	14 (93.3)	11 (73.3)	Reservation
A5-13: Use of physical education plant and equipment	Disagreement	15 (100)	9 (60)	Remove
A5-14: Utilization and creation of movement environments	Disagreement	15 (100)	9 (60)	Remove
A5-15: Measurement and evaluation of young children’s physical health	Agreement	15 (100)	11 (73.3)	Reservation
B. Curriculum design	Agreement	15 (100)	11 (73.3)	Reservation
B1. Type of course	Agreement	13 (86.7)	10 (66.7)	Reservation
B1-1: Rhythmic activities	Agreement	13 (86.7)	9 (60)	Reservation
B1-2: Sports program category	Agreement	14 (93.3)	9 (60)	Reservation
B1-3: Sports games category	Agreement	14 (93.3)	10 (66.7)	Reservation
B1-4: Functional exercises	Agreement	14 (93.3)	9 (60)	Reservation
B2. Course objectives	Agreement	15 (100)	12 (80)	Reservation
B2-1: Cognitive objective design	Agreement	15 (100)	11 (73.3)	Reservation
B2-2: Design of skill objectives	Agreement	15 (100)	11 (73.3)	Reservation
B2-3: Emotional objective design	Agreement	15 (100)	10 (66.7)	Reservation
B3. Teaching methods	Agreement	14 (93.3)	12 (80)	Reservation
B3-1. Direct teaching method	Agreement	14 (93.3)	10 (66.7)	Reservation
B3-2. Indirect teaching methods	Agreement	13 (86.7)	10 (66.7)	Reservation
B3-3. Situational teaching method	Agreement	15 (100)	9 (60)	Reservation
B3-4. Game teaching method	Agreement	15 (100)	9 (60)	Reservation
C. Curriculum implementation	Agreement	15 (100)	11 (73.3)	Reservation
C1. Course preparation	Agreement	15 (100)	9 (60)	Reservation
C1-1: Assessment of physical capabilities of young children	Agreement	15 (100)	10 (66.7)	Reservation
C1-2: Early childhood learning context creation	Agreement	15 (100)	10 (66.7)	Reservation
C2. Course organization	Agreement	15 (100)	12 (80)	Reservation
C2-1: Implementation of the curriculum plan	Disagreement	15 (100)	10 (66.7)	Remove
C2-2: Exercise equipment placement and smart use	Agreement	15 (100)	10 (66.7)	Reservation
C2-3: Teaching protection and help utilization	Agreement	14 (93.3)	11 (73.3)	Reservation
C2-4: Design and organization of competition activities	Agreement	14 (93.3)	10 (66.7)	Reservation
C2-5: Adjudication of disputes over competition activities	Agreement	14 (93.3)	10 (66.7)	Reservation
C3. Observational analysis	Agreement	15 (100)	12 (80)	Reservation
C3-1: Physical behavior in early childhood movement	Agreement	15 (100)	10 (66.7)	Reservation
C3-2: Psychological changes in early childhood exercise	Agreement	14 (93.3)	10 (66.7)	Reservation
C4. Language expression	Agreement	15 (100)	12 (80)	Remove
C4-1: Correctly describe motor skills	Disagreement	15 (100)	10 (66.7)	Remove
C4-2: Accurate instructional feedback and evaluation	Disagreement	15 (100)	11 (73.3)	Remove
C5. Movement demonstration	Agreement	15 (100)	13 (86.7)	Reservation
C5-1: Choosing the right time for demonstration	Agreement	13 (86.7)	12 (80)	Reservation
C5-2: Use of correct demonstration methods	Agreement	15 (100)	13 (86.7)	Reservation
C5-3: Make correct demonstration movements	Agreement	15 (100)	12 (80)	Reservation
C6. Classroom resilience	Agreement	15 (100)	12 (80)	Reservation
C6-1: Perception of potential exercise risks	Agreement	15 (100)	12 (80)	Reservation
C6-2: Critical incident response and management	Agreement	15 (100)	12 (80)	Reservation
D. Course evaluation	Agreement	14 (93.3)	10 (66.7)	Reservation
D1. Developmental evaluation of young children	Agreement	15 (100)	11 (73.3)	Reservation
D1-1: Evaluation of physical development of young children	Agreement	15 (100)	9 (60)	Reservation
D1-2: Evaluation of motor skills in young children	Agreement	15 (100)	12 (80)	Reservation
D2. Teacher growth evaluation	Agreement	15 (100)	10 (66.7)	Reservation
D2-1: Evaluation of achievement of instructional objectives	Agreement	15 (100)	9 (60)	Reservation
D2-2: Evaluation of satisfaction with teaching effectiveness	Agreement	15 (100)	8 (53.5)	Reservation
E. Research and innovation	Agreement	14 (93.3)	11 (73.3)	Reservation
E1. Research capacity	Agreement	15 (100)	10 (66.7)	Reservation
E1-1: Application of modern information technology	Agreement	14 (93.3)	8 (53.5)	Reservation
E1-2: Early childhood physical activity design and validation	Agreement	15 (100)	11 (73.3)	Reservation
E2. Innovative capacity	Agreement	15 (100)	9 (60)	Reservation
E2-1: Innovations in early childhood physical education teaching models	Agreement	15 (100)	9 (60)	Reservation
E2-2: Content innovation in early childhood physical education teaching and learning	Agreement	15 (100)	11 (73.3)	Reservation
E2-3: Innovations in early childhood physical education teaching methods	Agreement	15 (100)	10 (66.7)	Reservation

Regarding basic literacy (A), some experts believe that healthy behavior (A3) cannot support physical education competency goals, while believe that healthy behavior should be included as part of a structured early childhood physical education curriculum (Wang et al., 2020; Shi et al., 2025). Therefore, the healthy behavior indicator was retained for the second round of surveys. Experts believe that there is an overlap between health concepts (A3-1) and health habits (A3-2), so health habits (A3-2) have been removed and health concepts have been revised to health awareness.

The expert panel believes that sports knowledge (A5) includes the following content: Basic terminology in physical education (A5-1), Patterns of early childhood physical and Mental development (A5-3), Early childhood physical education values (A5-4), Early childhood physical education goals (A5-5), Early childhood physical education methods (A5-6), Early childhood physical education content (A5-7), Evaluation of early childhood physical education (A5-8), Making your own playthings and creating games (A5-11), Use of physical education plant and equipment (A5-13), Utilization and creation of movement environments (A5-14)has a repetitive relationship. For example, there is overlap in the content of early childhood physical education objectives (A5-5) and curriculum objectives (B2), early childhood physical education methods (A5-6) and teaching methods (B3), early childhood physical education content (A5-7) and motor skills (A2), and early childhood physical education assessment (A5-8) and developmental assessment of young children (D1). Therefore, delete the indicator: Basic Terms in Physical Education (A5-1). Patterns of early childhood physical and Mental development (A5-3), Early childhood physical education values (A5-4), Early childhood physical education goals (A5-5), Early childhood physical education methods (A5-6), Early childhood physical education content (A5-7), Evaluation of early childhood physical education (A5-8), Making your own playthings and creating games (A5-11), Use of physical education plant and equipment (A5-13), Utilization and creation of movement environments (A5-14),and change Characteristics of early childhood kinesiology (A5-2) to Theoretical knowledge of early childhood physical education.

Regarding course design (B), the expert panel found that there was a lack of comprehensiveness in course type (B1) and teaching methods (B3). Not only does it recommend increasing the number of sports-themed courses (B1-5) to achieve interdisciplinary integration, but it also proposes adding modern information technology teaching methods to enhance teachers’ digital education and teaching capabilities and digital sports literacy. In addition, it was considered that moral education objectives should be included in the course objectives (B2) because moral education can be achieved through cognitive objectives (B2-1), so the expert recommendation was not adopted.

Regarding course implementation (C), the expert panel believes that there is an overlap between course plan execution (C2-1) in course organization (C2) and course design (B), so (C2-1) has been removed. Since music and rhythm are essential elements of structured physical education courses for young children, audio and video processing and application (C2-5) have been added. Some experts believe that language expression (C4) in early childhood education should be used in a way that facilitates children’s understanding, and should not overemphasize professional motor skills and techniques. Accurate teaching feedback may be detrimental to understanding or cause children to lose interest. Therefore, language expression (C4) and its indicators should be removed.

Regarding course evaluation (D), because the expert panel believes that young children’s emotional development is an important indicator of social evaluation, an evaluation of young children’s emotional development (D1-3) has been added.

Regarding research and innovation (E). Because material resources are relatively scarce in relatively underdeveloped regions, the development and innovation of physical education curriculum resources for young children is very important for early childhood education. Therefore, innovation in physical education curriculum resources for young children (E2-4) has been added. After adding, removing, modifying survey indicators, 5 first-level indicators, 17 s-level indicators, and 54 third-level indicators were identified for the second round of surveys, as shown in Appendix 2.

### 3.2 Round two

In the second round of surveys, the experts and questions from the first round of Delphi research were included, and Cronbach’s alpha coefficient was (*α* = 0.977), indicating that the reliability of the second round of research tools was relatively high. We sent out a total of 15 invitations, and 15 experts participated in the survey, with an effective response rate of 100%. The expert panel’s judgment criteria were familiarity (Ca = 0.96), familiarity (Cs = 0.90), and authority (Cr = 0.93). Table 3 shows the results of the second round of the Delphi survey. The revised indicators Health Awareness (A3-2) and Early Childhood Physical Education Theory Knowledge (A5-1) achieved consensus (≥75%); the added indicators of Sports Theme Categories (B1-5), Information-Based Teaching Methods (B3-5), Audio-Visual Processing and Application (C2-6), Early Childhood Emotional Evaluation (D1-3), and Innovation in Early Childhood Physical Education Course Resources (E2-4) reached consensus (≥75%). After two rounds of Delphi surveys, the expert panel did not propose any further revisions. The five first-level indicators, 17 s-level indicators, and 54 third-level indicators in the physical education competency indicator system for early childhood teachers achieved good consistency and stability, and consensus was reached on all indicators.

**Table 3:** Results of round two of the Delphi study.

Indexes	Agreement or disagreement	Scores of 4 or 5	Scores of 5	Stability
A. Basic literacy	Agreement	14 (93.3)	11 (73.3)	Yes
A1. Physical fitness	Agreement	14 (93.3)	11 (73.3)	Yes
A1-1: Healthy fitness	Agreement	14 (93.3)	12 (80.0)	Yes
A1-2: Competitive fitness	Agreement	14 (93.3)	12 (80.0)	Yes
A2. Motor skills	Agreement	15 (100)	13 (86.7)	Yes
A2-1: Mobility skills	Agreement	14 (93.3)	12 (80.0)	Yes
A2-2: Manipulative skills	Agreement	14 (93.3)	12 (80.0)	Yes
A2-3: Stability skills	Agreement	14 (93.3)	9 (60.0)	Yes
A3. Health behaviors	Agreement	13 (86.7)	10 (66.7)	Yes
A3-1: Health awareness	Agreement	14 (93.3)	12 (80.0)	Yes
A3-2: Emotional control	Agreement	13 (86.7)	9 (60)	Yes
A4. Sportsmanship	Agreement	14 (93.3)	10 (66.7)	Yes
A4-1: Movement confidence	Agreement	14 (93.3)	11 (73.3)	Yes
A4-2: Follow the rules	Agreement	14 (93.3)	12 (80.0)	Yes
A4-3: Fair play	Agreement	14 (93.3)	11 (73.3)	Yes
A4-4: Solidarity	Agreement	14 (93.3)	11 (73.3)	Yes
A5. Sports science knowledge	Agreement	14 (93.3)	10 (66.7)	yes
A5-1: Theory of early childhood exercise science	Agreement	14 (93.3)	10 (66.7)	Yes
A5-2: Structured physical activity design	Agreement	14 (93.3)	11 (73.3)	Yes
A5-3: Interdisciplinary integrated curriculum design	Agreement	14 (93.3)	12 (80.0)	Yes
A5-4: Physical activity protection and treatment for young children	Agreement	15 (100)	12 (80.0)	Yes
A5-5: Physical fitness measurement and evaluation for young children	Agreement	15 (100)	9 (60.0)	Yes
B. Curriculum design	Agreement	15 (100)	12 (80.0)	Yes
B1. Type of course	Agreement	14 (93.3)	10 (66.7)	Yes
B1-1: Rhythmic activity category	Agreement	13 (86.7)	9 (60.0)	Yes
B1-2: Sports program category	Agreement	13 (86.7)	10 (66.7)	Yes
B1-3: Sports games category	Agreement	13 (86.7)	11 (73.7)	Yes
B1-4: Functional exercise category	Agreement	13 (86.7)	7 (46.7)	Yes
B1-5: Sports-themed category	Agreement	13 (86.7)	8 (53.3)	N/A
B2. Course objectives	Agreement	15 (100)	11 (73.3)	Yes
B2-1: Cognitive objective	Agreement	15 (100)	12 (80.0)	Yes
B2-2: Skill objectives	Agreement	15 (100)	12 (80.0)	Yes
B2-3: Emotional objective	Agreement	15 (100)	10 (66.7)	Yes
B3. Teaching methods	Agreement	15 (100)	12 (80.0)	Yes
B3-1: Direct teaching method	Agreement	15 (100)	11 (73.3)	Yes
B3-2: Indirect teaching methods	Agreement	15 (100)	9 (60.0)	Yes
B3-3: Situational teaching method	Agreement	15 (100)	10 (66.7)	Yes
B3-4: Game-based pedagogy	Agreement	15 (100)	10 (66.7)	N/A
B3-5: Informationization teaching method	Agreement	15 (100)	10 (66.7)	N/A
C. Curriculum implementation	Agreement	15 (100)	13 (86.7)	Yes
C1. Course preparation	Agreement	15 (100)	12 (80.0)	Yes
C1-1: Assessment of physical abilities of young children	Agreement	15 (100)	9 (60.0)	Yes
C1-2: Early childhood learning scenario creation	Agreement	15 (100)	12 (80.0)	Yes
C2. Course organization	Agreement	15 (100)	13 (86.7)	Yes
C2-1: Deployment and effective use of sports equipment	Agreement	15 (100)	12 (80.0)	Yes
C2-2: Teaching protection and assistance application	Agreement	15 (100)	9 (60.0)	Yes
C2-3: Competition activity design and organization	Agreement	15 (100)	10 (66.7)	Yes
C2-4: Adjudication of disputes arising from competitive activities	Agreement	15 (100)	9 (60.0)	Yes
C2-5: Audio and video processing and applications	Agreement	15 (100)	9 (60.0)	N/A
C3. Observational analysis	Agreement	15 (100)	11 (73.3)	Yes
C3-1: Physical behavior in early childhood exercise	Agreement	15 (100)	10 (66.7)	Yes
C3-2: Psychological changes in early childhood physical activity	Agreement	15 (100)	10 (66.7)	Yes
C4. Movement demonstration	Agreement	15 (100)	9 (60.0)	Yes
C4-1: Choosing right the time for demonstration	Agreement	15 (100)	13 (86.7)	Yes
C4-2: Use correct of demonstration methods	Agreement	15 (100)	12 (80.0)	Yes
C4-3: Make correct demonstration movements	Agreement	15 (100)	11 (73.3)	Yes
C5. Classroom resilience	Agreement	15 (100)	10 (66.7)	Yes
C5-1: Perception of potential exercise risks	Agreement	15 (100)	10 (66.7)	Yes
C5-2: Emergency response and handling	Agreement	15 (100)	12 (80.0)	Yes
D. Course evaluation	Agreement	15 (100)	10 (66.7)	Yes
D1. Developmental evaluation of young children	Agreement	15 (100)	12 (80.0)	Yes
D1-1: Evaluation of physical development of young children	Agreement	15 (100)	10 (66.7)	Yes
D1-2: Evaluation of motor skills in young children	Agreement	15 (100)	12 (80.0)	Yes
D1-3: Early childhood emotional assessment	Agreement	15 (100)	10 (66.7)	N/A
D2. Teacher growth evaluation	Agreement	14 (93.3)	11 (73.3)	Yes
D2-1: Evaluation of achievement of instructional objectives	Agreement	15 (100)	10 (66.7)	Yes
D2-2: Teaching evaluation of satisfaction with effectiveness	Agreement	15 (100)	10 (66.7)	Yes
E. Research and innovation	Agreement	14 (93.3)	10 (66.7)	Yes
E1. Research capacity	Agreement	15 (100)	12 (80.0)	Yes
E1-1: Application of modern information technology	Agreement	15 (100)	11 (73.3)	Yes
E1-2: Early childhood physical activity design and validation	Agreement	15 (100)	11 (73.3)	Yes
E2. Innovative capacity	Agreement	15 (100)	13 (86.7)	Yes
E2-1: Innovations in early childhood physical education teaching models	Agreement	15 (100)	10 (66.7)	Yes
E2-2: Content innovation in early childhood physical education teaching and learning	Agreement	15 (100)	11 (73.3)	Yes
E2-3: Early childhood physical education teaching innovations in methods	Agreement	15 (100)	12 (80.0)	Yes
E2-4: Innovations in early childhood physical education curriculum resources	Agreement	15 (100)	9 (60.0)	N/A

### 3.3 Indicator weights

After verification, the consistency ratio (CR) of the judgment matrix for the weight values of each level of indicators was less than 0.001, indicating that the hierarchical ranking of indicators and the combined weights are reasonable. Therefore, the weight values of the physical education ability indicators for preschool teachers can be calculated using the constructed judgment matrix. The weight values of each level of indicators are shown in Table 4.

**Table 4:** Weighting values for early childhood teachers’ physical education competency indicators.

Level 1 indicators	Weighting values	Secondary indicators	Weighting values	Tertiary indicators	Weighting values
A. Basic literacy	0.26946	A1. Physical fitness	0.29053	A1-1: Healthy Fitness	0.37722
				A1-2: Competitive fitness	0.62278
		A2. Motor skills	0.1708	A2-1: Mobility skills	0.49048
				A2-2: Manipulative skills	0.3119
				A2-3: Stability Skills	0.19762
		A3. Health behaviors	0.22802	A3-1: Health awareness	0.67535
				A3-2: Emotional control	0.32465
		A4. Sportsmanship	0.18361	A4-1: Movement confidence	0.26394
				A4-2: Follow the rules	0.20029
				A4-3: Fair play	0.16308
				A4-4: Solidarity	0.37269
		A5. Sports science knowledge	0.12704	A5-1: Theory of early childhood exercise science	0.39392
				A5-2: Structured physical activity design	0.19752
				A5-3: Interdisciplinary integrated curriculum design	0.17437
				A5-4: Physical activity protection and treatment for young children	0.11939
				A5-5: Physical fitness measurement and evaluation for young children	0.1148
B. Curriculum design	0.2865	B1. Type of course	0.17485	B1-1: Rhythmic activity category	0.1778
				B1-2: Sports program category	0.20212
				B1-3: Sports games category	0.13982
				B1-4: Functional exercise category	0.17227
				B1-5: Sports-themed category	0.30799
		B2. Course objectives	0.74355	B2-1: Cognitive objective	0.25006
				B2-2: Skill objectives	0.64524
				B2-3: Emotional objective	0.1047
		B3. Teaching methods	0.0816	B3-1: Direct teaching method	0.14807
				B3-2: Indirect teaching methods	0.21936
				B3-3: Situational teaching method	0.22109
				B3-4: Game-based pedagogy	0.22184
				B3-5: Informationization teaching method	0.18964
C. Curriculum implementation	0.21528	C1. Course preparation	0.27084	C1-1: Assessment of physical abilities of young children	0.696
				C1-2: Early childhood learning scenario creation	0.304
		C2. Course organization	0.30651	C2-1: Deployment and effective use of sports equipment	0.35508
				C2-2: Teaching protection and assistance application	0.18426
				C2-3: Competition Activity Design and Organization	0.10516
				C2-4: Adjudication of disputes arising from competitive activities	0.19848
				C2-5: Audio and video processing and applications	0.15702
		C3. Observational analysis	0.15775	C3-1: Physical behavior in early childhood exercise	0.55751
				C3-2: Psychological changes in early childhood physical activity	0.44249
		C4. Movement demonstration	0.17283	C4-1: Choosing right the time for demonstration	0.19972
				C4-2: Use correct of demonstration methods	0.36945
				C4-3: Make correct demonstration movements	0.43083
		C5. Classroom resilience	0.09207	C5-1: Perception of potential exercise risks	0.40946
				C5-2: Emergency Response and Handling	0.59054
D. Course evaluation	0.10711	D1. Developmental evaluation of young children	0.80833	D1-1: Evaluation of physical development of young children	0.41591
				D1-2: Evaluation of motor skills in young children	0.46352
				D1-3: Early childhood emotional assessment	0.12057
		D2. Teacher growth evaluation	0.19167	D2-1: Evaluation of achievement of instructional objectives	0.36901
				D2-2: Teaching evaluation of satisfaction with effectiveness	0.63099
E. Research and innovation	0.12165	E1. Research capacity	0.59573	E1-1: Application of modern information technology	0.58102
				E1-2: Early childhood physical activity design and validation	0.41898
		E2. Innovative capacity	0.40427	E2-1: Innovations in early childhood physical education teaching models	0.33219
				E2-2: Content innovation in early childhood physical education teaching and learning	0.40869
				E2-3: Early childhood physical education teaching innovations in methods	0.11146
				E2-4: Innovations in early childhood physical education curriculum resources	0.14766

## 4 Discussion

This study focuses on enhancing the practical capabilities of structured early childhood physical education programs. It employs an interdisciplinary approach to construct an evaluation framework for early childhood educators’ physical education competencies, laying the groundwork for the implementation of interdisciplinary “Physical Education + X” curriculum development. The survey results show that consensus (≧75%) was reached on all 5 first-level indicators, 17 s-level indicators, and 54 third-level indicators, and that stability was good. Therefore, the physical education competency indicators for preschool teachers developed in this study are consistent with the value orientation of structured preschool physical education curricula, providing a basis for the high-quality development of preschool physical education curricula. Regarding the modification of indicators, explanations that facilitate understanding have already been provided in the first round of survey results. Here, we will only discuss their reliability and rationality.

Reliability analysis of the physical education competency index system for preschool teachers. The Delphi method is currently the most effective method for evaluating and selecting indicators, and that the selection of experts is key to expert consultation using the Delphi method, directly affecting the reliability of research results (Li et al., 2021). We analyzed the Cronbach’s alpha, expert authority, and consensus level of the research tools. Based on the professional characteristics of early childhood educators, we selected 15 experts in the fields of sports science, child education, and kindergarten management from six provinces and municipalities across the country. These experts have both rich theoretical knowledge and strong practical skills, and are highly representative. Research indicates that the two-round Delphi survey research tool is effective and reliable (*α* > 0.97), with expert judgment (>0.85), authority (>0.85), and familiarity (>0.85), and consistency (CR < 0.001). The consensus level for primary, secondary, and tertiary indicators exceeded 85%, and the expert panel reached a strong consensus. Therefore, the physical education competency indicator system for preschool teachers constructed in this study is reliable.

A Rationality Analysis of the Physical Education Competency Index System for Preschool Teachers. the key to determining whether an evaluation indicator system is scientific and reasonable lies in whether the weights of the indicators are reasonable (Wei and Tao, 2022). This study reveals significant differences in the importance placed on curriculum design, curriculum evaluation, and research and innovation.

Research findings that curriculum design (0.2865) ranks first in terms of weight value. This view is inconsistent with the notion that curriculum implementation is more important than curriculum design (Shao, 2021). Analysis suggests that structured curricula, due to their clearer objectives, are more conducive to promoting the integration of “Physical Education +X” interdisciplinary courses. This indicates that structured physical education programs for young children are more effective in promoting their health and holistic development, aligning with the design and implementation principles of interdisciplinary physical education and health curricula in elementary schools (Zhang, 2025).

Basic literacy (0.26946) ranks second in terms of weighting value. This indicates that adopting an interdisciplinary approach to studying structured physical education curricula for young children should not only incorporate theories of early childhood education, but also the content and scope of sports science. The indicators for physical fitness, motor skills, healthy behavior, sports ethics, and sports knowledge included in this study are basically consistent with the conclusions of studies on the physical education literacy of preschool teachers (Xu, 2023; Kaiheng, 2025).

Course implementation (0.21528) ranks third in terms of weighting. This is a key part of how preschool teachers do physical education, and it really affects how well kids grow up healthy. This study not only clarifies the role of preschool teachers in structured physical education courses for young children, but also proposes the competencies that should be possessed throughout the entire course implementation process. This is consistent with the research perspectives on the professional competence of physical education teachers for young children (Kaiheng, 2025), the physical education teaching competence of preschool teachers (Jin, 2022).

Research and innovation (0.12165) ranks fourth in terms of weighting. This view is inconsistent with the notion that course evaluation is more important than research innovation capabilities (Shao, 2021). Analysis suggests that young children have shorter attention spans and are often more interested in novel things. In the limited physical education resources and environment of kindergartens, research and innovation capabilities are particularly crucial. For instance, modifying practice themes, content, and methods aligns with the fundamental requirements of interdisciplinary integration. Furthermore, early childhood education must develop in tandem with socioeconomic progress. Early childhood educators should prioritize the application of modern information technology in teaching while innovating instructional content and practices based on children’s physical and psychological characteristics. They should continuously optimize teaching models and resources, aligning with the perspectives of Kaiheng (2025) and Gu and Xie (2024).

Course evaluation (0.10711) ranks last in terms of weighting. This is inconsistent with the view that course teaching evaluation is more important than basic physical literacy (Shao, 2021). Analysis suggests that due to factors such as the professional competence of early childhood educators, methods for assessing child development, and the physical and psychological characteristics of young children, quantitative evaluation methods are not universally applicable in developmental assessments of preschoolers. Instead, subjective diagnostic evaluations of children’s participation in physical activities predominate, aligning with the perspectives of Keyuan (2022) and Jin (2022). The physical fitness, motor skills, and health behaviors encompassed within fundamental physical literacy in this study represent the core of current physical education reform and are key to comprehensively restructuring and optimizing the physical education curriculum system.

This study, grounded in early childhood physical education curriculum teaching and practice, has developed a structured assessment tool for preschool physical education curricula tailored for early childhood educators. Compared to previous research models, this study adopts a more focused objective, directly targeting structured early childhood physical education curricula. It enhances the scientific rigor and practical applicability of the evaluation indicator system while placing greater emphasis on integrating contemporary developmental characteristics into structured early childhood physical education curricula. This indicators not only effectively enhances early childhood educators’ practical teaching capabilities in physical education but also lays a foundation for the innovative development of early childhood physical education curricula.

## 5 Conclusion

The indicator system of physical education competence of early childhood teachers constructed in this study based on the structured early childhood physical education curriculum building path is reasonable and reliable. This study not only effectively enhances the physical education teaching skills of preschool teachers but also provides a basis for the development and evaluation of structured physical education curricula for preschoolers.

## 6 Limitations

Although this study has established a consensus-based competency framework for early childhood physical education teachers by focusing on the teaching and implementation of structured physical education curricula for young children, this framework still requires systematic training grounded in concrete case studies and sustained long-term practice for teachers to fully master it. Given that experts come from diverse disciplinary backgrounds and professional roles, the physical education competency framework for early childhood educators may be subject to bias. This evaluation system helps enhance early childhood educators’ self-awareness and professional competence in physical education curricula. However, it lacks empirical validation in actual teaching environments, and its practical effectiveness in promoting the coordinated development of young children’s basic motor skills and physical fitness remains to be verified. In the future, we will conduct targeted teacher training, experimental interventions, and field testing based on research findings to evaluate the usability and effectiveness of this assessment system in measuring young children’s basic motor skills and physical fitness levels. This will advance the high-quality development of structured physical education programs for preschoolers aged 3–6.

## Data Availability

The raw data supporting the conclusions of this article will be made available by the authors, without undue reservation.
