# Supplementary material for: Research on the construction of an indicator system for physical education teaching abilities of preschool teachers
Source: Front Psychol. 2025 Dec 16;16:1674552. doi: 10.3389/fpsyg.2025.1674552 (PMC12750617; doi:10.3389/fpsyg.2025.1674552)
Supplement: Supplementary file 1 [file Table_1.DOC]

**Survey Questionnaire on the Comparison of Weighting Factors in the Physical Education Competency Index System for Preschool Teachers**

Dear Experts:

Hello! This study ultimately constructed an indicator system for preschool teachers' physical education capabilities consisting of five first-level indicators, 17 second-level indicators, and 54 third-level indicators.This survey aims to consult on the weighting values of the physical education competency indicator system for preschool teachers, with the goal of clarifying the relevance and effectiveness of the indicator system evaluation.Please select the option that best reflects your understanding of the importance of each indicator for evaluating the physical education capabilities of early childhood teachers. Your opinions will serve as an important basis for establishing the indicator system. Thank you very much for your help!

**Filling Instructions**

**1.Questionnaire structure**

This questionnaire consists of two parts: comparing the importance of evaluation dimensions and comparing the importance of evaluation indicators. Each part lists two dimensions or indicators on the left and right sides of the page for you to compare their differences in importance. The middle section contains options for comparing the importance of the items.

**2.Option Selection**

(1)If you believe that the dimensions or indicators on the left side are more important than those on the right side of the same row, please select the corresponding option from the 17 options. The higher the score corresponding to one side, the more important it is.

(2)If you believe that the dimensions or indicators on the right side are more important than those on the left side of the same row, please select the corresponding option from the 17 options. The higher the score corresponding to one side, the more important it is.

**3. Reference examples**

As shown in the table below, you believe that basic literacy is much more important than curriculum evaluation in the physical education competency indicators for early childhood teachers.

| **Indexes** | **9** | **8** | **7** | **6** | **5** | **4** | **3** | **2** | **1** | **2** | **3** | **4** | **5** | **6** | **7** | **8** | **9** | **Indexes** |
| --- | --- | --- | --- | --- | --- | --- | --- | --- | --- | --- | --- | --- | --- | --- | --- | --- | --- | --- |
| A. Basic literacy |  | √ |  |  |  |  |  |  |  |  |  |  |  |  |  |  |  | D.Course evaluation |

**Indicator Weight Comparison Evaluation Table**

Regarding the assessment of the weights of the first-level indicators: Please compare the importance of each element of the first-level indicators in pairs. The higher the score for the corresponding indicator, the more important the indicator.

| **Comparison of the importance of first-level indicators** | | | | | | | | | | | | | | | | | | |
| --- | --- | --- | --- | --- | --- | --- | --- | --- | --- | --- | --- | --- | --- | --- | --- | --- | --- | --- |
| First-level indicators | **9** | **8** | **7** | **6** | **5** | **4** | **3** | **2** | **1** | **2** | **3** | **4** | **5** | **6** | **7** | **8** | **9** | First-level indicators |
| A. Basic literacy |  |  |  |  |  |  |  |  |  |  |  |  |  |  |  |  |  | B. Curriculum design |
| A. Basic literacy |  |  |  |  |  |  |  |  |  |  |  |  |  |  |  |  |  | C.Curriculum implementation |
| A. Basic literacy |  |  |  |  |  |  |  |  |  |  |  |  |  |  |  |  |  | D.Course evaluation |
| A. Basic literacy |  |  |  |  |  |  |  |  |  |  |  |  |  |  |  |  |  | E. Research and innovation |
| B. Curriculum design |  |  |  |  |  |  |  |  |  |  |  |  |  |  |  |  |  | C.Curriculum implementation |
| B. Curriculum design |  |  |  |  |  |  |  |  |  |  |  |  |  |  |  |  |  | D.Course evaluation |
| B. Curriculum design |  |  |  |  |  |  |  |  |  |  |  |  |  |  |  |  |  | E. Research and innovation |
| C.Curriculum implementation |  |  |  |  |  |  |  |  |  |  |  |  |  |  |  |  |  | D.Course evaluation |
| C.Curriculum implementation |  |  |  |  |  |  |  |  |  |  |  |  |  |  |  |  |  | E. Research and innovation |
| D.Course evaluation |  |  |  |  |  |  |  |  |  |  |  |  |  |  |  |  |  | E. Research and innovation |

Regarding the assessment of secondary indicator weights: Please compare the importance of each element of the secondary indicators in pairs. The higher the score for the corresponding indicator, the more important the indicator is.

| **Comparison of the importance of secondary indicators under the primary indicator “A. Basic literacy”** | | | | | | | | | | | | | | | | | | |
| --- | --- | --- | --- | --- | --- | --- | --- | --- | --- | --- | --- | --- | --- | --- | --- | --- | --- | --- |
| Second-level indicators | **9** | **8** | **7** | **6** | **5** | **4** | **3** | **2** | **1** | **2** | **3** | **4** | **5** | **6** | **7** | **8** | **9** | Second-level indicators |
| A1. Physical fitness |  |  |  |  |  |  |  |  |  |  |  |  |  |  |  |  |  | A2. Motor skills |
| A1. Physical fitness |  |  |  |  |  |  |  |  |  |  |  |  |  |  |  |  |  | A3. Health behaviors |
| A1. Physical fitness |  |  |  |  |  |  |  |  |  |  |  |  |  |  |  |  |  | A4. Sportsmanship |
| A1. Physical fitness |  |  |  |  |  |  |  |  |  |  |  |  |  |  |  |  |  | A5. Sports science knowledge |
| A2. Motor skills |  |  |  |  |  |  |  |  |  |  |  |  |  |  |  |  |  | A3. Health behaviors |
| A2. Motor skills |  |  |  |  |  |  |  |  |  |  |  |  |  |  |  |  |  | A4. Sportsmanship |
| A2. Motor skills |  |  |  |  |  |  |  |  |  |  |  |  |  |  |  |  |  | A5. Sports science knowledge |
| A3. Health behaviors |  |  |  |  |  |  |  |  |  |  |  |  |  |  |  |  |  | A4. Sportsmanship |
| A3. Health behaviors |  |  |  |  |  |  |  |  |  |  |  |  |  |  |  |  |  | A5. Sports science knowledge |
| A4. Sportsmanship |  |  |  |  |  |  |  |  |  |  |  |  |  |  |  |  |  | A5. Sports science knowledge |
| **Comparison of the importance of secondary indicators under the primary indicator “B. Course Design”** | | | | | | | | | | | | | | | | | | |
| Second-level indicators | **9** | **8** | **7** | **6** | **5** | **4** | **3** | **2** | **1** | **2** | **3** | **4** | **5** | **6** | **7** | **8** | **9** | Second-level indicators |
| B1. Type of course |  |  |  |  |  |  |  |  |  |  |  |  |  |  |  |  |  | B2. Course objectives |
| B1. Type of course |  |  |  |  |  |  |  |  |  |  |  |  |  |  |  |  |  | B3. Teaching methods |
| B2. Course objectives |  |  |  |  |  |  |  |  |  |  |  |  |  |  |  |  |  | B3. Teaching methods |
| **Comparison of the importance of secondary indicators under the primary indicator “C. Course Implementation”** | | | | | | | | | | | | | | | | | | |
| Second-level indicators | **9** | **8** | **7** | **6** | **5** | **4** | **3** | **2** | **1** | **2** | **3** | **4** | **5** | **6** | **7** | **8** | **9** | Second-level indicators |
| C1. Course preparation |  |  |  |  |  |  |  |  |  |  |  |  |  |  |  |  |  | C2. Course organization |
| C1. Course preparation |  |  |  |  |  |  |  |  |  |  |  |  |  |  |  |  |  | C3. Observational analysis |
| C1. Course preparation |  |  |  |  |  |  |  |  |  |  |  |  |  |  |  |  |  | C4. Movement demonstration |
| C1. Course preparation |  |  |  |  |  |  |  |  |  |  |  |  |  |  |  |  |  | C5.Classroom resilience |
| C2. Course organization |  |  |  |  |  |  |  |  |  |  |  |  |  |  |  |  |  | C3. Observational analysis |
| C2. Course organization |  |  |  |  |  |  |  |  |  |  |  |  |  |  |  |  |  | C4. Movement demonstration |
| C2. Course organization |  |  |  |  |  |  |  |  |  |  |  |  |  |  |  |  |  | C5.Classroom resilience |
| C3. Observational analysis |  |  |  |  |  |  |  |  |  |  |  |  |  |  |  |  |  | C4. Movement demonstration |
| C3. Observational analysis |  |  |  |  |  |  |  |  |  |  |  |  |  |  |  |  |  | C5.Classroom resilience |
| C4. Movement demonstration |  |  |  |  |  |  |  |  |  |  |  |  |  |  |  |  |  | C5.Classroom resilience |
| **Comparison of the importance of secondary indicators under the primary indicator “D. Course Evaluation”** | | | | | | | | | | | | | | | | | | |
| Second-level indicators | **9** | **8** | **7** | **6** | **5** | **4** | **3** | **2** | **1** | **2** | **3** | **4** | **5** | **6** | **7** | **8** | **9** | Second-level indicators |
| D1. Developmental evaluation of young  children |  |  |  |  |  |  |  |  |  |  |  |  |  |  |  |  |  | D2. Teacher growth evaluation |
| **Comparison of the importance of secondary indicators under the primary indicator “E. Research and Innovation”** | | | | | | | | | | | | | | | | | | |
| Second-level indicators | **9** | **8** | **7** | **6** | **5** | **4** | **3** | **2** | **1** | **2** | **3** | **4** | **5** | **6** | **7** | **8** | **9** | Second-level indicators |
| E1. Research capacity |  |  |  |  |  |  |  |  |  |  |  |  |  |  |  |  |  | E2. Innovative capacity |

Regarding the assessment of tertiary indicator weights: Please compare the importance of each element of the tertiary indicators in pairs. The higher the assessment score on one side, the more important the indicator .

| **Comparison of the importance of tertiary indicators under the secondary indicator “A1. Physical fitness”** | | | | | | | | | | | | | | | | | | |
| --- | --- | --- | --- | --- | --- | --- | --- | --- | --- | --- | --- | --- | --- | --- | --- | --- | --- | --- |
| Third-level indicators | **9** | **8** | **7** | **6** | **5** | **4** | **3** | **2** | **1** | **2** | **3** | **4** | **5** | **6** | **7** | **8** | **9** | Third-level indicators |
| A1-1:Healthy Fitness |  |  |  |  |  |  |  |  |  |  |  |  |  |  |  |  |  | A1-2:Competitive fitness |
| **Comparison of the importance of tertiary indicators under the secondary indicator “A2. Motor skills”** | | | | | | | | | | | | | | | | | | |
| Third-level indicators | **9** | **8** | **7** | **6** | **5** | **4** | **3** | **2** | **1** | **2** | **3** | **4** | **5** | **6** | **7** | **8** | **9** | Third-level indicators |
| A2-1: Mobility skills |  |  |  |  |  |  |  |  |  |  |  |  |  |  |  |  |  | A2-2: Manipulative skills |
| A2-1: Mobility skills |  |  |  |  |  |  |  |  |  |  |  |  |  |  |  |  |  | A2-3: Stability Skills |
| A2-2: Manipulative skills |  |  |  |  |  |  |  |  |  |  |  |  |  |  |  |  |  | A2-3: Stability Skills |
| **Comparison of the importance of tertiary indicators under the secondary indicator “A3. Healthy Behavior”** | | | | | | | | | | | | | | | | | | |
| Third-level indicators | **9** | **8** | **7** | **6** | **5** | **4** | **3** | **2** | **1** | **2** | **3** | **4** | **5** | **6** | **7** | **8** | **9** | Third-level indicators |
| A3-1: Health awareness |  |  |  |  |  |  |  |  |  |  |  |  |  |  |  |  |  | A3-2: Emotional control |
| **Comparison of the importance of tertiary indicators under the secondary indicator “A4. Physical Education and Moral Education”** | | | | | | | | | | | | | | | | | | |
| Third-level indicators | **9** | **8** | **7** | **6** | **5** | **4** | **3** | **2** | **1** | **2** | **3** | **4** | **5** | **6** | **7** | **8** | **9** | Third-level indicators |
| A4-1: Movement confidence |  |  |  |  |  |  |  |  |  |  |  |  |  |  |  |  |  | A4-2: Follow the rules |
| A4-1: Movement confidence |  |  |  |  |  |  |  |  |  |  |  |  |  |  |  |  |  | A4-3: Fair play |
| A4-1: Movement confidence |  |  |  |  |  |  |  |  |  |  |  |  |  |  |  |  |  | A4-4：Solidarity |
| A4-2: Follow the rules |  |  |  |  |  |  |  |  |  |  |  |  |  |  |  |  |  | A4-3: Fair play |
| A4-2: Follow the rules |  |  |  |  |  |  |  |  |  |  |  |  |  |  |  |  |  | A4-4：Solidarity |
| A4-3: Fair play |  |  |  |  |  |  |  |  |  |  |  |  |  |  |  |  |  | A4-4：Solidarity |
| **Comparison of the importance of tertiary indicators under the secondary indicator “A5. Sports Knowledge”** | | | | | | | | | | | | | | | | | | |
| Third-level indicators | **9** | **8** | **7** | **6** | **5** | **4** | **3** | **2** | **1** | **2** | **3** | **4** | **5** | **6** | **7** | **8** | **9** | Third-level indicators |
| A5-1: Theory of early childhood exercise science |  |  |  |  |  |  |  |  |  |  |  |  |  |  |  |  |  | A5-2: Structured physical activity design |
| A5-1: Theory of early childhood exercise science |  |  |  |  |  |  |  |  |  |  |  |  |  |  |  |  |  | A5-3: Interdisciplinary integrated curriculum design |
| A5-1: Theory of early childhood exercise science |  |  |  |  |  |  |  |  |  |  |  |  |  |  |  |  |  | A5-4: Physical activity protection and treatment for young children |
| A5-1: Theory of early childhood exercise science |  |  |  |  |  |  |  |  |  |  |  |  |  |  |  |  |  | A5-5: Physical fitness measurement and evaluation for young children |
| A5-2: Structured physical activity design |  |  |  |  |  |  |  |  |  |  |  |  |  |  |  |  |  | A5-3: Interdisciplinary integrated curriculum design |
| A5-2: Structured physical activity design |  |  |  |  |  |  |  |  |  |  |  |  |  |  |  |  |  | A5-4: Physical activity protection and treatment for young children |
| A5-2: Structured physical activity design |  |  |  |  |  |  |  |  |  |  |  |  |  |  |  |  |  | A5-5: Physical fitness measurement and evaluation for young children |
| A5-3: Interdisciplinary integrated curriculum design |  |  |  |  |  |  |  |  |  |  |  |  |  |  |  |  |  | A5-4: Physical activity protection and treatment for young children |
| A5-4: Physical activity protection and treatment for young children |  |  |  |  |  |  |  |  |  |  |  |  |  |  |  |  |  | A5-5: Physical fitness measurement and evaluation for young children |
| **Comparison of the importance of tertiary indicators under the secondary indicator “B1. Course categories”** | | | | | | | | | | | | | | | | | | |
| Third-level indicators | **9** | **8** | **7** | **6** | **5** | **4** | **3** | **2** | **1** | **2** | **3** | **4** | **5** | **6** | **7** | **8** | **9** | Third-level indicators |
| B1-1: Rhythmic activity category |  |  |  |  |  |  |  |  |  |  |  |  |  |  |  |  |  | B1-2: Sports program category |
| B1-1: Rhythmic activity category |  |  |  |  |  |  |  |  |  |  |  |  |  |  |  |  |  | B1-3: Sports games category |
| B1-1: Rhythmic activity category |  |  |  |  |  |  |  |  |  |  |  |  |  |  |  |  |  | B1-4: Functional exercise category |
| B1-1: Rhythmic activity category |  |  |  |  |  |  |  |  |  |  |  |  |  |  |  |  |  | B1-5: Sports-themed category |
| B1-2: Sports program category |  |  |  |  |  |  |  |  |  |  |  |  |  |  |  |  |  | B1-3: Sports games category |
| B1-2: Sports program category |  |  |  |  |  |  |  |  |  |  |  |  |  |  |  |  |  | B1-4: Functional exercise category |
| B1-2: Sports program category |  |  |  |  |  |  |  |  |  |  |  |  |  |  |  |  |  | B1-5: Sports-themed category |
| B1-3: Sports games category |  |  |  |  |  |  |  |  |  |  |  |  |  |  |  |  |  | B1-4: Functional exercise category |
| B1-3: Sports games category |  |  |  |  |  |  |  |  |  |  |  |  |  |  |  |  |  | B1-5: Sports-themed category |
| B1-4: Functional exercise category |  |  |  |  |  |  |  |  |  |  |  |  |  |  |  |  |  | B1-5: Sports-themed category |
| **Comparison of the importance of tertiary indicators under the secondary indicator “B2. Course objectives”** | | | | | | | | | | | | | | | | | | |
| Third-level indicators | **9** | **8** | **7** | **6** | **5** | **4** | **3** | **2** | **1** | **2** | **3** | **4** | **5** | **6** | **7** | **8** | **9** | Third-level indicators |
| B2-1: Cognitive objective |  |  |  |  |  |  |  |  |  |  |  |  |  |  |  |  |  | B2-2: Skill objectives |
| B2-1: Cognitive objective |  |  |  |  |  |  |  |  |  |  |  |  |  |  |  |  |  | B2-3: Emotional objective |
| B2-2: Skill objectives |  |  |  |  |  |  |  |  |  |  |  |  |  |  |  |  |  | B2-3: Emotional objective |
| **Comparison of the importance of tertiary indicators under the secondary indicator “B3. Teaching Methods”** | | | | | | | | | | | | | | | | | | |
| Third-level indicators | **9** | **8** | **7** | **6** | **5** | **4** | **3** | **2** | **1** | **2** | **3** | **4** | **5** | **6** | **7** | **8** | **9** | Third-level indicators |
| B3-1: Direct teaching method |  |  |  |  |  |  |  |  |  |  |  |  |  |  |  |  |  | B3-2: Indirect teaching methods |
| B3-1: Direct teaching method |  |  |  |  |  |  |  |  |  |  |  |  |  |  |  |  |  | B3-3: Situational teaching method |
| B3-1: Direct teaching method |  |  |  |  |  |  |  |  |  |  |  |  |  |  |  |  |  | B3-4: Game-based pedagogy |
| B3-1: Direct teaching method |  |  |  |  |  |  |  |  |  |  |  |  |  |  |  |  |  | B3-5: Informationization teaching method |
| B3-2: Indirect teaching methods |  |  |  |  |  |  |  |  |  |  |  |  |  |  |  |  |  | B3-3: Situational teaching method |
| B3-2: Indirect teaching methods |  |  |  |  |  |  |  |  |  |  |  |  |  |  |  |  |  | B3-4: Game-based pedagogy |
| B3-2: Indirect teaching methods |  |  |  |  |  |  |  |  |  |  |  |  |  |  |  |  |  | B3-5: Informationization teaching method |
| B3-3: Situational teaching method |  |  |  |  |  |  |  |  |  |  |  |  |  |  |  |  |  | B3-4: Game-based pedagogy |
| B3-3: Situational teaching method |  |  |  |  |  |  |  |  |  |  |  |  |  |  |  |  |  | B3-5: Informationization teaching method |
| B3-4: Game-based pedagogy |  |  |  |  |  |  |  |  |  |  |  |  |  |  |  |  |  | B3-5: Informationization teaching method |
| **Comparison of the importance of tertiary indicators under the secondary indicator “C1. Course Preparation”** | | | | | | | | | | | | | | | | | | |
| Third-level indicators | **9** | **8** | **7** | **6** | **5** | **4** | **3** | **2** | **1** | **2** | **3** | **4** | **5** | **6** | **7** | **8** | **9** | Third-level indicators |
| C1-1: Assessment of physical abilities of young children |  |  |  |  |  |  |  |  |  |  |  |  |  |  |  |  |  | C1-2: Early childhood learning  scenario creation |
| **Comparison of the importance of tertiary indicators under the secondary indicator “C2. Course Organization”** | | | | | | | | | | | | | | | | | | |
| Third-level indicators | **9** | **8** | **7** | **6** | **5** | **4** | **3** | **2** | **1** | **2** | **3** | **4** | **5** | **6** | **7** | **8** | **9** | Third-level indicators |
| C2-1: Deployment and effective use of sports equipment |  |  |  |  |  |  |  |  |  |  |  |  |  |  |  |  |  | C2-2: Teaching protection and assistance application |
| C2-1: Deployment and effective use of sports equipment |  |  |  |  |  |  |  |  |  |  |  |  |  |  |  |  |  | C2-3: Competition Activity Design and Organization |
| C2-1: Deployment and effective use of sports equipment |  |  |  |  |  |  |  |  |  |  |  |  |  |  |  |  |  | C2-4: Adjudication of disputes arising from competitive activities |
| C2-1: Deployment and effective use of sports equipment |  |  |  |  |  |  |  |  |  |  |  |  |  |  |  |  |  | C2-5: Audio and video processing and applications |
| C2-2: Teaching protection and assistance application |  |  |  |  |  |  |  |  |  |  |  |  |  |  |  |  |  | C2-3: Competition Activity Design and Organization |
| C2-2: Teaching protection and assistance application |  |  |  |  |  |  |  |  |  |  |  |  |  |  |  |  |  | C2-4: Adjudication of disputes arising from competitive activities |
| C2-2: Teaching protection and assistance application |  |  |  |  |  |  |  |  |  |  |  |  |  |  |  |  |  | C2-5: Audio and video processing and applications |
| C2-3: Competition Activity Design and Organization |  |  |  |  |  |  |  |  |  |  |  |  |  |  |  |  |  | C2-4: Adjudication of disputes arising from competitive activities |
| C2-3: Competition Activity Design and Organization |  |  |  |  |  |  |  |  |  |  |  |  |  |  |  |  |  | C2-5: Audio and video processing and applications |
| C2-4: Adjudication of disputes arising from competitive activities |  |  |  |  |  |  |  |  |  |  |  |  |  |  |  |  |  | C2-5: Audio and video processing and applications |
| **Comparison of the importance of tertiary indicators under the secondary indicator “C3. Observation and Analysis”** | | | | | | | | | | | | | | | | | | |
| Third-level indicators | **9** | **8** | **7** | **6** | **5** | **4** | **3** | **2** | **1** | **2** | **3** | **4** | **5** | **6** | **7** | **8** | **9** | Third-level indicators |
| C3-1: Physical behavior in early childhood exercise |  |  |  |  |  |  |  |  |  |  |  |  |  |  |  |  |  | C3-2: Psychological changes in early childhood physical activity |
| **Comparison of the importance of tertiary indicators under the secondary indicator “C5. Movement Demonstration”** | | | | | | | | | | | | | | | | | | |
| Third-level indicators | **9** | **8** | **7** | **6** | **5** | **4** | **3** | **2** | **1** | **2** | **3** | **4** | **5** | **6** | **7** | **8** | **9** | Third-level indicators |
| C4-1: Choosing right the time for demonstration |  |  |  |  |  |  |  |  |  |  |  |  |  |  |  |  |  | C4-2: Use correct of demonstration methods |
| C4-1: Choosing right the time for demonstration |  |  |  |  |  |  |  |  |  |  |  |  |  |  |  |  |  | C4-3: Make correct demonstration movements |
| C4-2: Use correct of demonstration methods |  |  |  |  |  |  |  |  |  |  |  |  |  |  |  |  |  | C4-3: Make correct demonstration  movements |
| **Comparison of the importance of tertiary indicators under the secondary indicator “C6. Classroom Adaptability”** | | | | | | | | | | | | | | | | | | |
| Third-level indicators | **9** | **8** | **7** | **6** | **5** | **4** | **3** | **2** | **1** | **2** | **3** | **4** | **5** | **6** | **7** | **8** | **9** | Third-level indicators |
| C5-1: Perception of potential exercise risks |  |  |  |  |  |  |  |  |  |  |  |  |  |  |  |  |  | C5-2: Emergency Response and Handling |
| **Comparison of the importance of tertiary indicators under the secondary indicator “D1. Developmental evaluation of young children”** | | | | | | | | | | | | | | | | | | |
| Third-level indicators | **9** | **8** | **7** | **6** | **5** | **4** | **3** | **2** | **1** | **2** | **3** | **4** | **5** | **6** | **7** | **8** | **9** | Third-level indicators |
| D1-1: Evaluation of physical development of young children |  |  |  |  |  |  |  |  |  |  |  |  |  |  |  |  |  | D1-2: Evaluation of motor skills in young children |
| D1-1: Evaluation of physical development of young children |  |  |  |  |  |  |  |  |  |  |  |  |  |  |  |  |  | D1-3: Early childhood emotional emotional assessment |
| D1-2: Evaluation of motor skills in young children |  |  |  |  |  |  |  |  |  |  |  |  |  |  |  |  |  | D1-3: Early childhood emotional emotional assessment |
| **Comparison of the importance of tertiary indicators under the secondary indicator “D2. Growth-oriented evaluation of teachers”** | | | | | | | | | | | | | | | | | | |
| Third-level indicators | **9** | **8** | **7** | **6** | **5** | **4** | **3** | **2** | **1** | **2** | **3** | **4** | **5** | **6** | **7** | **8** | **9** | Third-level indicators |
| D2-1: Evaluation of achievement of instructional objectives |  |  |  |  |  |  |  |  |  |  |  |  |  |  |  |  |  | D2-2: Teaching evaluation of satisfaction with effectiveness |
| **Comparison of the importance of tertiary indicators under the secondary indicator “E1. Research Capability”** | | | | | | | | | | | | | | | | | | |
| Third-level indicators | **9** | **8** | **7** | **6** | **5** | **4** | **3** | **2** | **1** | **2** | **3** | **4** | **5** | **6** | **7** | **8** | **9** | Third-level indicators |
| E1-1: Application of modern information technology |  |  |  |  |  |  |  |  |  |  |  |  |  |  |  |  |  | E1-2: Early childhood physical activity design and validation |
| **Comparison of the importance of tertiary indicators under the secondary indicator “E2. Innovation Capability”** | | | | | | | | | | | | | | | | | | |
| Third-level indicators | **9** | **8** | **7** | **6** | **5** | **4** | **3** | **2** | **1** | **2** | **3** | **4** | **5** | **6** | **7** | **8** | **9** | Third-level indicators |
| E2-1: Innovations in early childhood physical education teaching models |  |  |  |  |  |  |  |  |  |  |  |  |  |  |  |  |  | E2-2: Content innovation in early childhood physical education teaching and learning |
| E2-1: Innovations in early childhood physical education teaching models |  |  |  |  |  |  |  |  |  |  |  |  |  |  |  |  |  | E2-3: Early childhood physical education teaching innovations in methods |
| E2-1: Innovations in early childhood physical education teaching models |  |  |  |  |  |  |  |  |  |  |  |  |  |  |  |  |  | E2-4: Innovations in early childhood physical education curriculum resources |
| E2-2: Content innovation in early childhood physical education teaching and learning |  |  |  |  |  |  |  |  |  |  |  |  |  |  |  |  |  | E2-3: Early childhood physical education teaching innovations in methods |
| E2-2: Content innovation in early childhood physical education teaching and learning |  |  |  |  |  |  |  |  |  |  |  |  |  |  |  |  |  | E2-4: Innovations in early childhood physical education curriculum resources |
| E2-3: Early childhood physical education teaching innovations in methods |  |  |  |  |  |  |  |  |  |  |  |  |  |  |  |  |  | E2-4: Innovations in early childhood physical education curriculum resources |

This survey has ended. Thank you for your participation!
