# Supplementary material for: Research on the construction of an indicator system for physical education teaching abilities of preschool teachers
Source: Front Psychol. 2025 Dec 16;16:1674552. doi: 10.3389/fpsyg.2025.1674552 (PMC12750617; doi:10.3389/fpsyg.2025.1674552)
Supplement: Supplementary file 3 [file Table_3.DOCX]

**Expert on the System of Physical Education Competency Indicators for Early Childhood Teachers Panel Questionnaire (Round 2)**

**Respected experts:**

Hello! I am a physical education teacher in Chongqing preschool education college, and I am doing a research on the construction of the index system of physical education competence of early childhood teachers. The purpose of this study is to provide theoretical basis and practical support for the cultivation of physical education competence of early childhood teachers in China, in order to promote the high-quality development of preschool education professional training. After the first round of surveys and interviews, the research of this project has made good progress, but consensus cannot be well reached on some indicators. In this round of survey, we added, deleted, modified and merged some indicators according to the opinions of the first round of expert group, enriched the connotation of the indicators, and determined 5first-level indicators, 17second-level indicators and 54 third-level indicators after optimization. Please fill in the questionnaire again according to your teaching experience. Thank you for participating in the survey in your busy schedule, and we sincerely appreciate your support and cooperation!

**List of indicator systems for physical education competencies of early childhood teachers**

| **Level 1 indicators (5)** | **Secondary indicators**  **(17)** | | **Tertiary indicators**  **(54)** |  |
| --- | --- | --- | --- | --- |
| A. Basic literacy | A1. Physical fitness | | A1-1:Healthy Fitness |  |
|  |  |  | A1-2:Competitive fitness |  |
|  | A2. Motor skills | A2-1: Mobility skills | |  |
|  |  | A2-2: Manipulative skills | |  |
|  |  | A2-3: Stability Skills | |  |
|  | A3. Health behaviors | A3-1: Health awareness | |  |
|  |  | A3-2: Emotional control | |  |
|  | A4. Sportsmanship | A4-1: Movement confidence | |  |
|  |  | A4-2: Compliance | |  |
|  |  | A4-3: Fair play | |  |
|  |  | A4-4: Solidarity | |  |
|  | A5. Sports science knowledge | A5-1: Theory of early childhood exercise science | |  |
|  |  | A5-2: Structured physical activity design | |  |
|  |  | A5-3: Interdisciplinary integrated curriculum design | |  |
|  |  | A5-4: Physical activity protection and treatment for young children | |  |
|  |  | A5-5: Physical fitness measurement and evaluation for young children | |  |
| B. Curriculum design | | B1. course Type of | B1-1: Rhythmic activity category | |
|  |  |  | B1-2: Sports program category | |
|  |  |  | B1-3: Sports games category | |
|  |  |  | B1-4: Functional exercise category | |
|  |  |  | B1-5: Sports-themed category | |
|  |  | B2. Course objectives | B2-1: Cognitive objective | |
|  |  |  | B2-2: Skill objectives | |
|  |  |  | B2-3: Emotional objective | |
|  |  | B3. Teaching methods | B3-1: Direct teaching method | |
|  |  |  | B3-2: Indirect teaching methods | |
|  |  |  | B3-3: Situational teaching method | |
|  |  |  | B3-4: Game-based pedagogy | |
|  |  |  | B3-5: Informationization teaching method | |
| C.Curriculum implementation | | C1. Course preparation | C1-1: Assessment of physical abilities of young children | |
|  |  |  | C1-2: Early childhood learning  scenario creation | |
|  |  | C2. Course organization | C2-1: Deployment and effective use of sports equipment | |
|  |  |  | C2-2: Teaching protection and assistance application | |
|  |  |  | C2-3: Competition Activity Design and Organization | |
|  |  |  | C2-4: Adjudication of disputes arising from competitive activities | |
|  |  |  | C2-5: Audio and video processing and  applications | |
|  |  | C3. Observational analysis | C3-1: Physical behavior in early childhood exercise | |
|  |  |  | C3-2: Psychological changes in early childhood physical activity | |
|  |  | C4. Movement demonstration | C4-1: Choosing right the time for  demonstration | |
|  |  |  | C4-2: Use correct of demonstration  methods | |
|  |  |  | C4-3: Make correct demonstration  movements | |
|  |  | C5. Classroom resilience | C5-1: Perception of potential exercise risks | |
|  |  |  | C5-2: Emergency Response and Handling | |
| D.Course  evaluation | | D1. Developmental evaluation of young  children | D1-1: Evaluation of physical  development of young children | |
|  |  |  | D1-2: Evaluation of motor skills in  young children | |
|  |  |  | D1-3: Early childhood emotional  emotional assessment | |
|  |  | D2. Teacher growth evaluation | D2-1: Evaluation of achievement of instructional objectives | |
|  |  |  | D2-2: Teaching evaluation of satisfaction with effectiveness | |
| E. Research and innovation | | E1. Research capacity | E1-1: Application of modern information technology | |
|  |  |  | E1-2: Early childhood physical activity design and validation | |
|  |  | E2. Innovative capacity | E2-1: Innovations in early childhood  physical education teaching models | |
|  |  |  | E2-2: Content innovation in early childhood physical education teaching and learning | |
|  |  |  | E2-3: Early childhood physical education teaching innovations in methods | |
|  |  |  | E2-4: Innovations in early childhood physical education curriculum resources | |

**Part I Basic information**

1.Your gender?

○Male ○ Female

2.What is your education level?

○Ph.D. ○ M.S. ○ Undergraduate

3.The nature of your workplace?

○Higher Education ○ Kindergarten ○ Education and Training

4.What is your area of specialization?

○Physical Education ○ Preschool Education ○ Other

5.The number of years you have been engaged in your professional field?

○1-5 years ○ 6-10 years ○ 11-15 years ○ 15+ years

# Part II Scale for assessing the reasonableness of indicators

## **Instructions for Completion:** This questionnaire adopts a five-point Likert scale, in which the degree of reasonableness of each indicator is categorized into five grades: Very Reasonable (5 points), Reasonable (4 points), General (3 points), Unreasonable (2 points) and Very Unreasonable (1 point). Please read carefully and judge the degree of reasonableness of each indicator and put a tick on the corresponding value. If you think there are indicators or other comments that have not been taken into account, please add or supplement the indicators at the end of the column and judge their degree of reasonableness. Example:

| **Level 1 indicators** | **Secondary indicators** | **Explanation of the meaning of secondary indicators** | **Judgment of**  **Reasonableness** | | | | |  |
| --- | --- | --- | --- | --- | --- | --- | --- | --- |
|  |  |  | **5** | **4** | **3** | **2** | **1** |  |
| A.Basic literacy | A1.Physical fitness | The ability of early childhood teachers to meet the needs of daily life, outside the organization of teaching activities, have the spare capacity to enjoy leisure activities and be able to adapt to unexpected situations is divided into health fitness and competitive fitness. For example, body composition, strength,speed,endurance, and balance. | √ |  |  |  |  |  |
|  | A2.Motor skills | Basic motor skills (e.g., walking, running, jumping, pushing, pulling, grasping, kicking, throwing, etc.) and basic motor skills (e.g., track and field, gymnastics, ballroom sports, etc.) to meet the demands of regular physical education for teachers of young children in daily activities |  | √ |  |  |  |  |
|  | A3.Health behaviors | Behaviors of to enhance physical fitness and maintain physical and mental health , including adequate sleep, balanced nutrition, and exercise habits. early childhood teachers daily in activities |  |  |  | √ |  |  |
| **Your comments and suggestions:**  (Additional/supplemental indicators) as ...... , therefore it is proposed to add/supplement the indicator......(Other suggestions) I think the indicator is justified on a scale of 5/4/3/2/1. (Other suggestions) I think .... | | | | | | | | |

**Assessment of the degree of legitimacy of the first-tier indicators**

**Guidelines for :** completion What ? the follow in g are the education competencies physical for early childhood teachers Can level 1 indicators reasonably cover the five physical education competencies of early childhood teachers?

| **Level 1 indicators** | **Explanation of the connotations of level 1 indicators** | **Judgment of**  **Reasonableness** | | | | |
| --- | --- | --- | --- | --- | --- | --- |
|  |  | **5** | **4** | **3** | **2** | **1** |
| A. Basic literacy | The exhibited emotions, attitudes, knowledge, values, and unique qualities and behaviors in sports and physical education , which include not only physical fitness and motor skills in the activities of daily life and teaching early childhood teachers, etc., but also individual factors such as , and values.thoughts, behaviors |  |  |  |  |  |
| B. Curriculum design | Early childhood teachers should have the ability to design a curriculum that encompasses curriculum objectives, content, and structured physical activity the design of methods of organization and implementation |  |  |  |  |  |
| C. Curriculum implementation | Early childhood teachers should have the ability to that includes implement and manage a program structured physical activity explanation and demonstration, practice, and other implementation components. |  |  |  |  |  |
| D. Course evaluation | Early childhood teachers should have the ability to evaluate the curriculum during , including the kindergarten physical education day subjective and objective evaluation and goal attainment. |  |  |  |  |  |
| E. Research and innovation | Early childhood teachers should have the ability to research and innovate , including in content, mode and method in kindergarten one-day physical activities physical activity design and validation, and sports environment creation. |  |  |  |  |  |
| **Your comments and suggestions:**  (Additional/supplemental indicators) as ...... , therefore it is proposed to add/supplement the indicator ..... (Other suggestions) I think the indicator is justified on a scale of 5/4/3/2/1. (Other suggestions) I think .... | | | | | | |

# Judging the degree of legitimacy of secondary indicators

**Guidelines for :** Completion check whether the secondary indicators included in the primary indicators are comprehensive and reasonable? Is clear? Is there any doubt about the meaning of the secondary indicators? The expression of the secondary indicator.

| **Level 1 indicators** | **Secondary indicators** | **Explanation of the meaning of secondary indicators** | | | **Judgment of Reasonableness** | | | | | | |
| --- | --- | --- | --- | --- | --- | --- | --- | --- | --- | --- | --- |
|  |  |  |  |  | **5** | **4** | | **3** | | **2** | **1** |
| A.Basic literacy | A1. Physical fitness | The ability of early childhood teachers to meet the needs of daily life, outside the organization of teaching activities, have the spare capacity to enjoy leisure activities and be able to adapt to unexpected situations is divided into health fitness and competitive fitness. For example, body composition, strength, speed,endurance, and balance. | | |  |  | |  | |  |  |
|  | A2. Motor skills | Basic motor skills (including walking, running, jumping, pushing, pulling, grasping, kicking, throwing, etc.) and basic motor skills(including gymnastics, ball games, etc.) to meet the demands of regular physical education. | | |  |  | |  | |  |  |
|  | A3. Health behaviors | Behaviors of to enhance physical fitness and maintain physical and mental health , including adequate sleep, balanced nutrition, and exercise habits. early childhood teachers daily in activities. | | |  |  | |  | |  |  |
|  | A4.  Sportsmanship | The behavioral norms, sports ethics as well as the values and spirituality that should be followed by early childhood teachers in sports, encompassing adherence to the rules, teamwork, and a correct view of winning and losing. | | |  |  | |  | |  |  |
|  | A5.Sports science knowledge | Early childhood teachers kindergarten one-day physical education activities should have theoretical knowledge, practical teaching experience and operational knowledge and skills , including knowledge of physical and mental developmental characteristics and sensitive periods of young children, prevention of common injuries and diseases, and structured early childhood physical education curriculum design. | | |  |  | |  | |  |  |
|  | Your comments and suggestions:  (Additional/supplemental indicators) as ...... , therefore it is proposed to add/supplement the indicator ..... (Other suggestions) I think the indicator is justified on a scale of 5/4/3/2/1. (Other suggestions) I think .... | | | | | | | | | | |
| B.Curriculum design | B1.Type of course | Meet the reserve capacity of kindergarten teachers to teach curriculum in kindergarten one-day physical activities, including rhythmic activities, physical games, sports programs, functional exercises, and thematic education categories. |  |  | | |  | |  | |  |
|  | B2. Course objectives | The goals that early childhood teachers need to achieve in a kindergarten physical activity day or in a particular curriculum design, including cognitive, skill,and affective goals. |  |  | | |  | |  | |  |
|  | B3.Teaching methods | Methods rationally used by early childhood teachers to achieve curriculum objectives and motor skills during the kindergarten physical activity day, including games, situational teaching, explanation, and practice. |  |  | | |  | |  | |  |
|  | Your comments and suggestions:  (Additional/supplemental indicators) as ...... , therefore it is  proposed to add/supplement the indicator ..... (Other suggestions) I  think the indicator is justified on a scale of 5/4/3/2/1. (Other suggestions) I think .... | | | | | | | | | | |
| C.Curriculum implementation | C1. Course preparation | Early childhood teachers in the kindergarten day sports activities in order to guide children to acquire good sports habits or sports interest in the means used, such as: analysis of the learning situation, experience preparation, context creation, story introduction and material preparation. |  |  | | |  | |  | |  |
|  | C2. Course organization | The ability of early childhood teachers to organize the teaching and learning process, and to guide and interact with play activities in order to achieve the curriculum objectives during the kindergarten physical activity day. |  |  | | |  | |  | |  |
|  | C3.Observational analysis | The analysis and feedback ability of the teaching problems that kindergarten teachers should have in order to achieve the curriculum objectives in the kindergarten day physical activities, including behavioral analysis, psychological analysis and reflection ability. |  |  | | |  | |  | |  |
|  | C4.Movement demonstration | Early childhood teachers should have the ability to model movements, including modeling timing and placement, in order to achieve the curriculum objectives during the kindergarten physical activity day. |  |  | | |  | |  | |  |
|  | C5.  Classroom resilience | The classroom resilience that kindergarten teachers should have in order to achieve the curriculum objectives during the kindergarten physical education day includes the handling of emergencies, teaching resilience,and so on. |  |  | | |  | |  | |  |
|  | **Your comments and suggestions:**  (Additional/supplemental indicators) as ...... , therefore it is proposed to add/supplement the indicator ..... (Other suggestions) I think the indicator is justified on a scale of 5/4/3/2/1. (Other suggestions) I think .... | | | | | | | | | | |
| D. Course  evaluation | D1.Early childhood developmental assessment | The subjective and objective evaluation skills that kindergarten teachers should have in the development of young children in order to achieve the curriculum objectives during the kindergarten day's physical education activities include the reasonable evaluation of the strength of the upper limbs, good and bad motor skills, and the strengths and weaknesses of the emotions and feelings. |  |  | | |  | |  | |  |
|  | D2. Teacher growth evaluation | Self-assessment and reflection skills that early childhood teachers should have in their own professional development in order to achieve curriculum goals during the kindergarten physical activity day. |  |  | | |  | |  | |  |
|  | **Your comments and suggestions:**  (Additional/supplemental indicators) as ...... , therefore it is proposed to add/supplement the indicator ..... (Other suggestions) I think the indicator is justified on a scale of 5/4/3/2/1. (Other suggestions) I think .... | | | | | | | | | | |
| E. Research  and innovation | E1.Research  capacity | Early childhood teachers should  have identify, problems in order to  expand their teaching resources during the kindergarten physical education day, the ability to solve and validate e.g., curriculum program design and validation. |  |  | | |  | |  | |  |
|  |  |  |  |  | | |  | |  | |  |
|  | E2.  Innovative capacity | In order to expand the teaching resources in kindergarten one-day physical activities, early childhood teachers should have the mode, content, innovation, including of method and Resources the development of garden-based curriculum and the construction of teaching resource base. |  |  | | |  | |  | |  |
|  | **Your comments and suggestions:**  (Additional/supplemental indicators) as ...... , therefore it is proposed to add/supplement the indicator ..... (Other suggestions) I think the indicator is justified on a scale of 5/4/3/2/1. (Other suggestions) I think .... | | | | | | | | | | |

# Review of the rationality of the three-tier indicators

**Guidelines for completing the form:** Is the relationship between the primary - secondary - tertiary indicators reasonable? Are the tertiary indicators included in the secondary indicators comprehensive and reasonable? Is the expression of tertiary indicators clear? Is there any doubt about the meaning of the tertiary indicators?

Level 1 indicators: A. Basic literacy

| **Secondary indicators** | **Tertiary indicators** | **Interpretation of the three levels of indicators** | **Judgment of**  **Reasonableness** | | | | |
| --- | --- | --- | --- | --- | --- | --- | --- |
|  |  |  | **5** | **4** | **3** | **2** | **1** |
| A1. Physical fitness | A1-1: Healthy fitness | Individuals have sufficient energy to engage in daily work (study) without fatigue, and at the same time have spare capacity to enjoy recreational and leisure activities, as well as the ability to adapt to unexpected situations. |  |  |  |  |  |
|  | A1-2: Competitive fitness | The basic qualities that maintain the body's ability to move, e.g., strength, speed, endurance, etc. |  |  |  |  |  |
|  | **Your comments and suggestions:**  (Additional/supplementary indicators) due to ...... , therefore it is proposed to add/supplement the indicator ..... (Other suggestions) I think the indicator is justified on a scale of 5/4/3/2/1. (Other suggestions) I think .... | | | | | | |
| A2. Motor  skills | A2-1: Mobility  skills | The ability to move the body from one  position to another,e.g., walking, crossing. |  |  |  |  |  |
|  | A2-2:  Manipulative skills | The ability of the body to move by controlling some instrument, e.g., hitting a ball. |  |  |  |  |  |
|  | A2-3:  Stability Skills | The ability to control , e.g., flexion, suspension.the body stability in order to maintain during movement. |  |  |  |  |  |
|  | **Your comments and suggestions:**  (Additional/supplementary indicators) due to ...... , therefore it is proposed to add/supplement the indicator ..... (Other suggestions) I think the indicator is justified on a scale of 5/4/3/2/1. (Other suggestions) I think .... | | | | | | |
| A3. Health  behaviors | A3-1: Health  awareness | Early childhood teachers are equipped  with the cognition of exercise for health  and basic knowledge of exercise hygiene and health, equipped with conscious health self-management skills and sensitivity to value communication.such as:exercise can strengthen the body,the principle of hydration in small  amounts and many times. |  |  |  |  |  |
|  | A3-2: Emotional control | Early childhood educator are able to maintain a positive and optimistic emotional state during physical activity and are able to redirect negative emotions, e.g., by turning frustration into motivation. |  |  |  |  |  |
|  | **Your comments and suggestions:**  (Additional/supplementary indicators) due to ...... , therefore it is proposed to add/supplement the indicator ..... (Other suggestions) I think the indicator is justified on a scale of 5/4/3/2/1. (Other suggestions) I think .... | | | | | | |
| A4.Sportsmanship | A4-1: Movement  confidence | Kindergarten teacher is able to defy the odds and persevere through the challenges of the  sport. |  |  |  |  |  |
|  | A4-2: Follow the rules | Kindergarten teachers are able to take the  initiative to understand the rules of physical activity and follow them consciously. |  |  |  |  |  |
|  | A4-3: Fair Play | Early childhood teachers are able to insist on fair play in sports and have a proper outlook on winning and losing. |  |  |  |  |  |
|  | A4-4:Solidarity | Kindergarten teachers are willing to actively participate in group-based activities and collaborate on tasks in the movement. |  |  |  |  |  |
|  | **Your comments and suggestions:**  (Additional/supplementary indicators) due to ...... , therefore it is proposed to add/supplement the indicator ..... (Other suggestions) I think the indicator is justified on a scale of 5/4/3/2/1. (Other suggestions) I think .... | | | | | | |
|  | A5-1: Theory of early childhood exercise science | Early childhood teachers understand the basic structure and physiological functions of young children's body systems, and the characteristics of young children's physical activity, physical fitness and motor skills, in order to scientifically protect and teach young children in physical activities. |  |  |  |  |  |
|  | A5-2: Structured physical activity design | Early childhood teachers are able to design and create curricula based on children's physical and mental developmental patterns and motor skills, as well as the philosophy of the school,e.g.,movement-themed curricula. |  |  |  |  |  |
|  | A5-3: Interdisciplinary integrated curriculum design | Early childhood teachers are able to incorporate knowledge from areas multiple subject for use in physical education activities,e.g.,incorporating in physical education activities knowledge of .aesthetics and moral education. |  |  |  |  |  |
|  | A5-4: Physical activity protection and treatment for young children | Kindergarten teachers are able to reasonably avoid risk factors and correctly handle accidents during physical activities, e.g., applying pressure to stop bleeding and bandaging wounds. |  |  |  |  |  |
|  | A5-5: Physical fitness measurement and evaluation for young children | Early childhood teachers are able to correctly grasp the methods and criteria for measuring and evaluating the physical fitness of young children. |  |  |  |  |  |
|  | **Your comments and suggestions:**  (Additional/supplemental indicators) as , therefore it is proposed  to add/supplement the indicator ..... (Other suggestions) I think the indicator is justified on a scale of 5/4/3/2/1. (Other suggestions) I think .... | | | | | | |
| B1.  Type of course | B1-1: Rhythmic  Activity category | Early childhood teachers are able to conduct physical activities for young children with rhythmic movements such as morning exercises and gymnastics. |  |  |  |  |  |
|  | B1-2: Sports  program  category | Early childhood teachers are able to conduct physical activities for young children in sports such as basketball and soccer. |  |  |  |  |  |
|  | B1-3: Sports  games category | Early childhood teachers are able to conduct physical activities for young children in the form of ball games and traditional folk games. |  |  |  |  |  |
|  | B1-4:  Functional  exercise  category | Early childhood teachers are able to with the conduct physical activities for young children goal of developing basic motor skills |  |  |  |  |  |
|  | B1-5: Sports-themed  category | Early childhood teachers are able to  in an design thematic education programs  interdisciplinary and integrated manner for  early childhood physical and health education activities. |  |  |  |  |  |
|  | **Your comments and suggestions:**  (Additional/supplementary indicators) due to ...... , therefore it is proposed to add/supplement the indicator ..... (Other suggestions) I think the indicator is justified on a scale of 5/4/3/2/1. (Other suggestions) I think .... | | | | | | |
| B2. Course  objectives | B2-1: Cognitive  objective | Early childhood teachers understand the keys to cognitive goal design, e.g., cognitive-life integration. |  |  |  |  |  |
|  | B2-2: Skill  objectives | Early childhood teachers understand the keys to skill goal design, e.g., combining movement and play. |  |  |  |  |  |
|  | B2-3: Emotional  objective | Early childhood teachers understand  the keys to designing emotional goals, such  as: play and emotional experiences. |  |  |  |  |  |
|  | **Your comments and suggestions:**  (Additional/supplementary indicators) due to ...... , therefore it is proposed to add/supplement the indicator ..... (Other suggestions) I think the indicator is justified on a scale of 5/4/3/2/1. (Other suggestions) I think .... | | | | | | |
| B3. Teaching  methods | B3-1.Direct  teaching method | Early childhood teachers are able to correctly select and when implementing Instructional activities.use direct teaching methods,such as:modeling,demonstration,and practice, |  |  |  |  |  |
|  | B3-2. Indirect  teaching  methods | Early childhood teachers are able to correctly select and when implementing Instructional activities.use indirect teaching methods such as exploration,explanation,and discussion. |  |  |  |  |  |
|  | B3-3.  Situational  teaching method | Early childhood teachers are able to reasonably create movement scenarios to achieve curriculum objectives when implementing teaching activities, e.g.,imitating a frog jumping on a lotus leaf to develop jumping ability. |  |  |  |  |  |
|  | B3-4.Game  teaching method | Early childhood teachers are able to make reasonable .Choices when Implementing instructional activities in order about the use of game-based pedagogy to achieve curriculum goals. For example, relay games. |  |  |  |  |  |
|  | B3-5:  Informatization teaching method | Early childhood teachers are able to implement teaching and learning activities in a way that rationalizes the use of informational teaching methods to achieve the goals of the curriculum. |  |  |  |  |  |
|  | **Your comments and suggestions:**  (Additional/supplemental indicators) as , there fore it is proposed to add/supplement the indicator ..... (Other suggestions) I think the indicator is justified on a scale of 5/4/3/2/1. (Other suggestions) I think .... | | | | | | |
| C1. Course  preparation | C1-1: Assessment of physical abilities of young children | Early childhood teachers children's learning prior to the lesson assess , e.g., experience, ability. |  |  |  |  |  |
|  | C1-2: Early  childhood  learning  scenario  creation | Early childhood teachers create a Curriculum environment prior to the lesson to set the stage for implementing the content and to stimulate interest in participation.For example,choosing movement skills that with those that need to be mastered warm up by are positively migrating |  |  |  |  |  |
|  | **Your comments and suggestions:**  (Additional/supplemental indicators) as , therefore it is proposed to add/supplement the indicator ..... (Other suggestions) I think the indicator is justified on a scale of 5/4/3/2/1. (Other suggestions) I  think .... | | | | | | |
|  | C2-1:Deployment and effective use of sports equipment | Teachers of young children are able to maximize the value of exercise equipment in their activities and are able to guide children's thinking. |  |  |  |  |  |
|  | C2-2: Teaching protection and assistance application | Early childhood teachers are able to protect and assist vulnerable children during activities to complete movement exercises and avoid motor risks. |  |  |  |  |  |
| C2.Course organization | C2-3: Competition Activity Design and Organization | Early childhood teachers are able to design and organize various types of parent-child competitions in their activities. |  |  |  |  |  |
|  | C2-4:  Adjudication of disputes arising from competitive activities | Early childhood teachers are able to effectively resolve competition disputes and controversies in activities. |  |  |  |  |  |
|  | C2-5: Audio and video processing and applications | Early childhood teachers are able to effectively process the audio and video information needed in the program and implement effective interactions. |  |  |  |  |  |
|  | **Your comments and suggestions:**  (Additional/supplemental indicators) as , therefore it is proposed to add/supplement the indicator ..... (Other suggestions) I think the indicator is justified on a scale of 5/4/3/2/1. (Other suggestions) I think .... | | | | | | |
| C3.Observational analysis | C3-1: Physical  behavior in  early childhood  movement | Early childhood teachers are able to identify abnormal motor behavior problems during activities such as hyper mobility and delayed motor development . |  |  |  |  |  |
|  | C3-2:Psychological  changes in  early childhood  exercise | Early childhood teachers are able to during the activities determine the psychological changes of children and guide and adjust the contents in time. |  |  |  |  |  |
|  | **Your comments and suggestions:**  (Additional/supplementary indicators) due to ...... , therefore it is proposed to add/supplement the indicator ..... (Other suggestions) I think the indicator is justified on a scale of 5/4/3/2/1. (Other suggestions) I think .... | | | | | | |
| C4. Movement demonstration | C4-1: Choosing the right time for  demonstration | Early childhood teachers conduct physical activities with well-timed demonstrations that are easy for children to observe and learn from. |  |  |  |  |  |
|  | C4-2: Use  correct of demonstration methods | Early childhood teachers model physical activities in a way that is appropriate and easy for children to learn and practice. |  |  |  |  |  |
|  | C4-3: Make correct demonstration movements | Teachers of young children demonstrate correct movements and teach young children correct movements and postures during physical activities. |  |  |  |  |  |
|  | **Your comments and suggestions:**  (Additional/supplementary indicators) due to ...... , therefore it is proposed to add/supplement the indicator ..... (Other suggestions) I think the indicator is justified on a scale of 5/4/3/2/1. (Other suggestions) I think .... | | | | | | |
| C5.Classroom resilience | C5-1:  Perception of potential exercise risks | Early childhood teachers are able to avoid and determine sports when organizing physical Activities points of risk. |  |  |  |  |  |
|  | C5-2: Emergency response and handling | Early childhood teachers are able to deal with in a timely manner when organizing physical activities emergencies to avoid deterioration . |  |  |  |  |  |
|  | **Your comments and suggestions:**  (Additional/supplemental indicators) as , therefore it is proposed to add/supplement the indicator ..... (Other suggestions) I think the indicator is justified on a scale of 5/4/3/2/1. (Other suggestions) I think .... | | | | | | |
| D1.Developmental evaluation of young children | D1-1: Evaluation of physical  development of young children | Teachers of young children are able to assess the level of physical development of young children from motor performance, e.g., subjective assessment of physical fitness and form. |  |  |  |  |  |
|  | D1-2: Evaluation of motor skills in  young children | Teachers of young children are able to motor performance evaluate the level of motor skill development,e.g., from subjective judgments of whether or not movement is delayed. |  |  |  |  |  |
|  | D1-3: Early childhood emotional  emotional assessment | Teachers of young children are able to from motor performance accurately determine and evaluate children's emotional mood states , e.g., joy,fear, etc. |  |  |  |  |  |
|  | **Your comments and suggestions:**  (Additional/supplemental indicators) as , therefore it is proposed to add/supplement the indicator ..... (Other suggestions) I think the indicator is justified on a scale of 5/4/3/2/1. (Other suggestions) I think .... | | | | | | |
| D2. Teacher  growth evaluation | D2-1: Evaluation of achievement of instructional objectives | Early childhood teachers are able to evaluate the achievement of objectives of structured physical activities, facilitating timely adjustments to lesson objectives. |  |  |  |  |  |
|  | D2-2: Teaching evaluation of satisfaction with effectiveness | Early childhood teachers are able to evaluate the effectiveness of structured physical activity to enhance professional skills |  |  |  |  |  |
|  | **Your comments and suggestions:**  (Additional/supplemental indicators) as , therefore it is proposed to add/supplement the indicator ..... (Other suggestions) I think the indicator is justified on a scale of 5/4/3/2/1. (Other suggestions) I think .... | | | | | | |
| E1. Research  capacity | E1-1: Application of modern information technology | Early childhood teachers are able to moderate the application of modern information technology to assist in teaching and learning.For example,digital recording and analysis of sports data during physical activity. |  |  |  |  |  |
|  | E1-2: Early childhood physical activity design and validation | Early childhood teachers are able to physical activity identify, validate and solve problems . |  |  |  |  |  |
|  | **Your comments and suggestions:**  (Additional/supplemental indicators) as , therefore it is proposed to add/supplement the indicator ..... (Other suggestions) I think the indicator is justified on a scale of 5/4/3/2/1. (Other suggestions) I think .... | | | | | | |
| E2.Innovative capacity | E2-1: Early  childhood  physical  education  innovations in  models teaching | Early childhood teachers are able to implement teaching and learning activities models, e.g.,curriculum models integrated with new policies.in physical education with new |  |  |  |  |  |
|  | E2-2: Content  innovation in  early childhood  physical education  teaching and  learning | Early childhood teachers are able to in physical education with new teach activities content,e.g., folk games are introduced. |  |  |  |  |  |
|  | E2-3: Early  childhood  physical  education  teaching  innovations in  methods | Early childhood teachers are able to in new in physical activities utilize teaching resources ways , e.g., balloons for multiple uses. |  |  |  |  |  |
|  | E2-4:  Innovations in  early childhood  physical  education  curriculum  resources | Early childhood teachers are able to create new curriculum resources.through early childhood physical education activities |  |  |  |  |  |
|  | **Your comments and suggestions:**  (Additional/supplemental indicators) as , therefore it is proposed to add/supplement the indicator ..... (Other suggestions) I think the indicator is justified on a scale of 5/4/3/2/1. (Other suggestions) I think .... | | | | | | |

# Part III Quantification of the degree of authority of experts

**1.Instructions for filling in the form:** the degree of authority of an expert is generally determined and calculated by two indicators, namely, the basis of judgment made by the expert (Ca) and the expert's familiarity with the issue (Cs), and is usually by self-evaluation of differentiated grades, and each indicator is rated in terms of importance by using a large (3 points), a medium (2 points), and a small (1 point), so please make a judgment and then put

a "√".

**2.Basis of judgment (Ca) self-assessment scale Instructions for completing the form :** Please judge the basis of the level 1 indicators in this questionnaire and tick the appropriate option.

| **Level 1 indicators** | **Basis of judgment** | **Degree of importance** | | | | | |
| --- | --- | --- | --- | --- | --- | --- | --- |
|  |  | Important  (3 points) | | Moderate  (3 points) | | Unimportant  (3 points) | |
| **A.Basic literacy** | Practical experience |  | |  | |  | |
|  | Theoretical analysis |  | |  | |  | |
|  | Reference to domestic  and foreign sources/peer  understanding |  | |  | |  | |
|  | Intuition |  | |  | |  | |
| **B.Curriculum design** | Practical experience |  | |  | |  | |
|  | Theoretical analysis |  | |  | |  | |
|  | Reference to domestic  and foreign sources/peer  understanding |  | |  | |  | |
|  | Intuition |  | |  | |  | |
| **C.Curriculum implementation** | Practical experience |  | |  | |  | |
|  | Theoretical analysis |  | |  | |  | |
|  | Reference to domestic  and foreign sources/peer  understanding |  | |  | |  | |
|  | Intuition |  | |  | |  | |
| **D. Course evaluation** | Practical experience |  | |  | |  | |
|  | Theoretical analysis |  | |  | |  | |
|  | Reference to domestic  and foreign sources/peer  understanding |  | |  | |  | |
|  | Intuition |  | |  | |  | |
| **E.Research and innovation** | Practical experience |  | |  | |  | |
|  | Theoretical analysis | |  | |  | |  |
|  | Reference to domestic  and foreign sources/peer  understanding | |  | |  | |  |
|  | Intuition | |  | |  | |  |

## **3.Familiarity (Cs) self-assessment scale Instructions for filling in the form:** Please judge your in this questionnaire familiarity with the level 1 indicators and put the corresponding option a tick on . The of indicator degree of is divided into five grades: very familiarity each familiar (5 points), familiar (4 points), general (3 points), unfamiliar (2 points), very unfamiliar (1 point), please make your judgment and put the corresponding item a "√" .

| **Level 1 indicators** | **Familiarity** | | | | |
| --- | --- | --- | --- | --- | --- |
|  | **Very Familiar**  (5 points) | **Familiar**  (5 points) | **General**  (5 points) | **Unfamiliar**  (5 points) | **Very unfamiliar** (5 points) |
| **A. Basic literacy** |  |  |  |  |  |
| **B. Curriculum**  **design** |  |  |  |  |  |
| **C. Curriculum**  **implementation** |  |  |  |  |  |
| **D. Course evaluation** |  |  |  |  |  |
| **E. Research and**  **innovation** |  |  |  |  |  |

**Part IV Validity scale for the expert advice questionnaire**

Instructions for completing the questionnaire: Please judge the validity and accuracy of this questionnaire. Do you think this questionnaire is reasonable in terms of its structural design? Does the content of the questionnaire clearly express the theme of the questionnaire? What is the validity of the questionnaire? The degree of is categorized into five grades: very reasonableness of indicator each reasonable (5 points), reasonable (4 points), general (3 points), unreasonable (2 points), and very unreasonable (1 point), and put the corresponding option a tick on "√".

| **Questionnaire validity** | **Degree of reasonableness** | | | | |
| --- | --- | --- | --- | --- | --- |
|  | **Very reasonable** (5 points) | **Reasonable** (4 points) | **General** (3 points) | **Unreasonable** (2 points) | **Very unreasonable** (1 points) |
| **Structural validity** |  |  |  |  |  |
| **Content validity** |  |  |  |  |  |
| **Overall validity** |  |  |  |  |  |

This correspondence ends here, thank you again for your help; I wish you a happy life and a successful work!
